# Development and validation of prognostic nomograms for early-onset colon cancer in different tumor locations: a population-based study

**DOI:** 10.1186/s12876-023-02991-1

**Published:** 2023-10-21

**Authors:** Sirui Zhu, Jiawei Tu, Wei Pei, Zhaoxu Zheng, Jianjun Bi, Qiang Feng

**Affiliations:** Department of Colorectal Surgery, National Cancer Center/National Clinical Research Center for Cancer/Cancer Hospital, Chinese Academy of Medical Sciences and Peking Union Medical College, Beijing, China

**Keywords:** Nomogram, Early-onset colon cancer, Right-sided colon cancer, Left-sided colon cancer, SEER

## Abstract

**Objective:**

The prevalence of early-onset colon cancer (EOCC) among individuals below the age of 50 has shown a marked upward trend in recent years. The embryology, clinical symptoms, incidence, molecular pathways, and oncologic outcomes differ between right-sided and left-sided colon cancers. However, the differences have not been fully researched in EOCC. Our study aims to develop and validate prognostic nomograms predicting overall survival (OS) and cancer-specific survival (CSS) for EOCC in different tumor locations based on the Surveillance, Epidemiology, and End Results (SEER) database.

**Methods:**

Using the SEER database, a total of 5,588 patients with EOCC were extracted and divided into development and validation cohorts in a random allocation ratio of 7:3 across three groups. Univariate and multivariate Cox regression analyses were performed to identify independent prognostic factors influencing OS and CSS outcomes. These factors were then utilized to construct nomogram models. The prognostic capabilities of the three models were assessed through various evaluation metrics, including the concordance index (C-index), receiver operating characteristic (ROC) curves, calibration curves, decision curve analysis (DCA), and validation cohorts respectively. Additionally, survival curves of the low- and high-risk groups were calculated using the Kaplan–Meier method together with the log-rank test.

**Results:**

Significant differences in clinical features were observed between right-sided and left-sided EOCCs, particularly in terms of OS (52 months vs 54 months) as demonstrated by Kaplan–Meier curves. Transverse-sided EOCCs exhibited clinical characteristics similar to right-sided EOCCs, suggesting a potential shared tumor microenvironment and therapeutic considerations. Advanced stage, liver metastasis, poor grade, elevated pretreatment carcinoembryonic antigen (CEA) level, chemotherapy, and perineural invasion were identified as independent prognostic factors across all three tumor locations and were incorporated into the nomogram model. Nomograms were constructed to predict the probability of 3- and 5-year OS and CSS. The C-index and calibration plots showed that the established nomograms had good consistency between actual clinical observations and predicted outcomes. ROC curves with calculated area under the curve (AUC) values exceeded 0.8 for all three groups in both the development and validation cohorts, indicating robust predictive performance for OS and CSS. Furthermore, decision curve analysis (DCA) plots revealed a threshold probability range of 0.1 to 0.9, within which the nomogram model exhibited maximum benefit. Kaplan–Meier curves exhibited significant differences between the low- and high-risk groups in EOCC for all three tumor locations in OS and CSS, further validating the prognostic value of the nomogram models.

**Conclusions:**

We successfully developed three precise nomogram models for EOCCs in different tumor locations, providing valuable support for clinicians in guiding clinical treatments and facilitating further prospective follow-up studies.

**Supplementary Information:**

The online version contains supplementary material available at 10.1186/s12876-023-02991-1.

## Introduction

Colorectal cancer (CRC) has emerged as the third most common cancer and the second leading cause of cancer-related mortality worldwide [1]. Fortunately, the incidence and mortality rates of CRC have shown a decline in recent years due to the widespread adoption of colonoscopy screening and advancements in treatment [2]. However, there has been a concerning rise in the incidence of CRC in individuals below the age of 50, highlighting the emergence of early-onset colon cancer (EOCC) as a predominant contributor to this increase [3, 4]. Current literature suggests that EOCC is characterized by poorer tumor differentiation and more advanced disease at diagnosis compared to late-onset CRC [5]. Nevertheless, the development of dedicated diagnostic and therapeutic protocols specifically tailored for EOCC remains an unmet clinical need [6].

Embryologically, the colon exhibits a distinct right and left-sidedness as it differentiates from the midgut and hindgut.. he transverse colon, positioned at the boundary between the midgut and hindgut, lacks a definitive consensus on its origin and has often been excluded in many studies [7]. Depending on the location within the colon, colon cancer (CC) may exhibit different patterns of disease progression, cancer-specific survival (CSS) and overall survival (OS) [8]. Previous research has identified potential risk factors for CC, including pathological grade, surgery, and chemotherapy [9]. However, the specific risk factors associated with different tumor locations within the colon are still unclear and warrant further investigation.

The Surveillance, Epidemiology, and End Results (SEER) program, managed by the National Cancer Institute (NCI), serves as a valuable resource for epidemiological data on cancer incidence and survival rates in the United States [10]. Numerous studies have leveraged the SEER database to analyze clinical issues, thereby contributing to advancements in cancer treatment. Nomograms, widely utilized in medical studies, offer an intuitive and effective means of predicting outcomes. By assessing the length of each variable’s line in the nomogram, one can determine its impact on the overall prognosis.

Building upon these foundations, our study aims to develop prognostic nomograms that will furnish patients and clinicians with essential prognostic information and identify risk factors specific to EOCC in different tumor locations.

## Materials and methods

### Data collection and patient selection

The patients, diagnosed with EOCC, were carefully selected from the SEER Research Plus Data 22 registry spanning the years 2000 to 2019. In order to maintain consistency, our study focused solely on the 7th AJCC staging system. Therefore, we exclusively extracted data from the SEER database pertaining to EOCC, specifically covering the period from January 2010 to December 2016, enabling us to delve into survival rates and prognostic factors. To be included in our analysis, patients with EOCC needed to satisfy the following criteria: 1) their age fell between 18 and 50 years, and 2) the primary tumor was located in the colon, as indicated by the relevant codes C18.0, C18.2-C18.7. We defined tumors proximal to the hepatic flexure as right-sided tumors, tumors at transverse as transverse-sided tumors, and tumors distal to the splenic flexure as left-sided tumors.

Each patient’s comprehensive profile encompassed a range of demographic, clinical, pathological, and therapeutic variables. These variables comprised gender, age at the time of diagnosis, tumor size, tumor location, pathological stage, pathological grade, histological type, primary site surgery, regional lymph node dissection, chemotherapy, radiotherapy, presence of metastasis, pretreatment CEA levels, perineural invasion, and follow-up information. To ensure the reliability and validity of our analysis, all eligible patients were randomly allocated to two cohorts, adhering to a 7:3 ratio. We excluded patients who met any of the following criteria: (1) those with incomplete survival or follow-up data, (2) individuals diagnosed with multiple primary or secondary tumors,, and (3) those with either a survival time of zero or unavailable data (NA).

### Statistical analysis

Through the utilization of Cox regression models, we performed calculations to determine a 95% confidence interval (CI) and hazard ratio (HR). In order to identify potential prognostic factors, those showing significant differences in the univariate Cox regression analysis were further examined through multivariate analysis. Subsequently, utilizing the R software and based on the results of the multivariate analysis, we constructed and evaluated nomograms. These nomograms were designed to predict the 3-year and 5-year OS and CSS specifically for EOCC patients. The effectiveness of the novel nomograms was assessed using various methods, including the concordance index (C-index), time-dependent receiver operating characteristic (ROC) curve, and the area under the ROC curve (AUC). To compare the predicted survival rates from the nomograms with the actual survival rates, calibration curves were plotted. Additionally, the decision curve analysis (DCA) was performed to evaluate the clinical usefulness of the nomograms by quantifying the net benefits at different threshold probabilities.

To determine the optimal cut-off values, X-tile software (version 3.6.1) developed by Yale University in New Haven, CT, USA, was employed. Statistical analyses were conducted using R software (version 3.6.1, http://www.r-project.org/) and IBM SPSS software (version 25.0) by IBM in Armonk, NY, USA. Various R packages, including "rms," "survival," "magick," "timeROC," "ggplotify," and "cowplot," were utilized for the construction and assessment of the nomograms. A significance level of 0.05 was adopted, and any reported *p*-values below this threshold were considered statistically significant.

## Result

### Difference between various tumor location

**Fig. 1:**
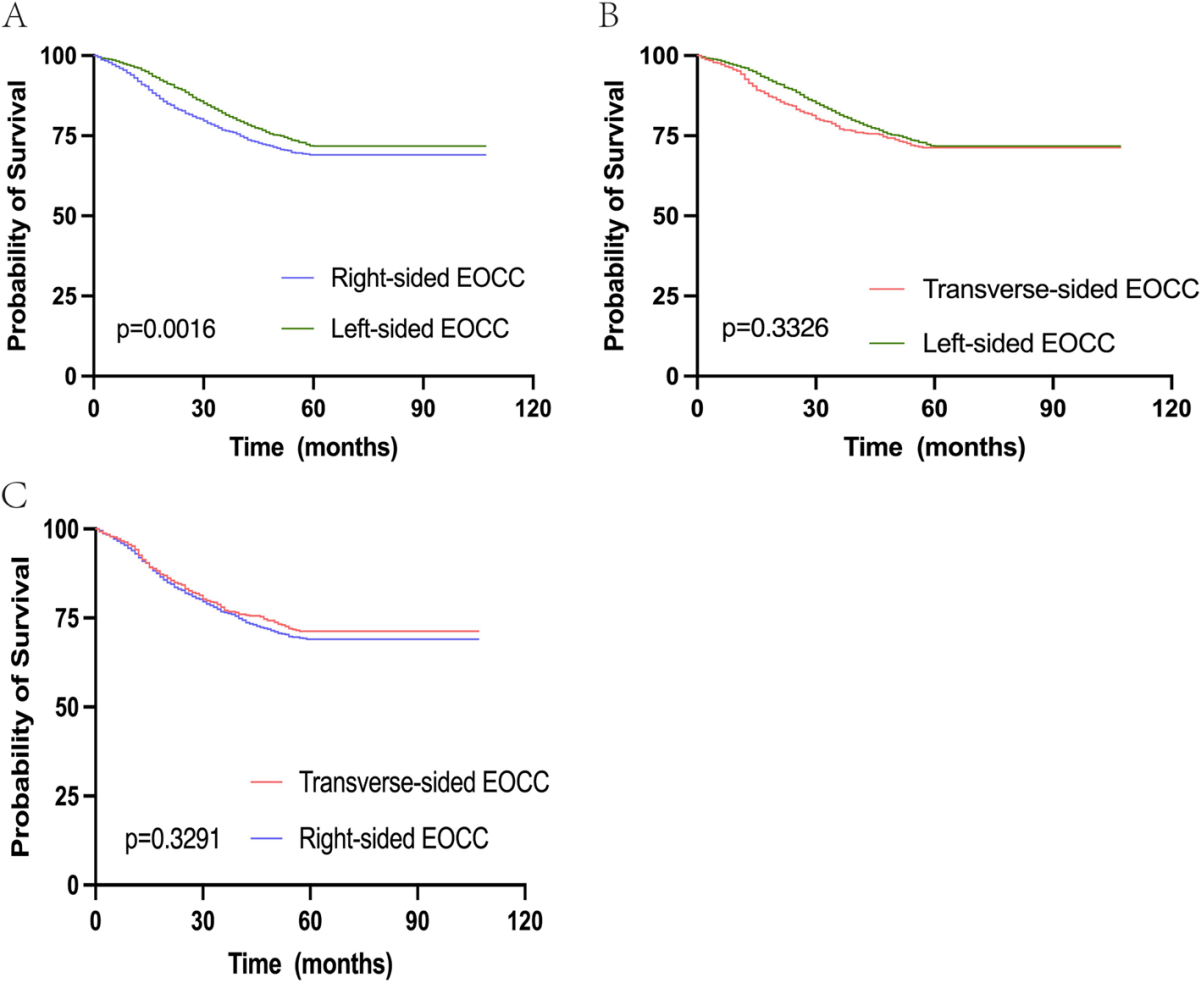
The Kaplan–Meier survival analysis in three different tumor locations. **A** The Kaplan–Meier survival analysis between right-sided EOCCs and left-sided EOCCs. **B** The Kaplan–Meier survival analysis between transverse-sided EOCCs. **C** The Kaplan–Meier survival analysis between transverse-sided EOCCS and right-sided EOCCs. Abbreviations: *EOCC* Early-onset colon cancer

The study enrolled a total of 1980 patients with right-sided EOCC (35.4%) and 3035 patients with left-sided EOCC (54.3%). Baseline data for these patients were obtained from the SEER database and are summarized in Table 1. Interestingly, left-sided EOCC showed a higher prevalence among females (*p* < 0.001), which contrasts with previous research findings [11]. Specific subtypes of adenocarcinoma, such as mucinous adenocarcinoma and signet ring cell carcinoma, were more commonly observed in right-sided EOCC (*p* < 0.001). Although right-sided EOCC exhibited deeper tumor penetration (*p* = 0.005) and larger tumor size (*p* < 0.001), a higher proportion of patients with left-sided EOCC developed distant metastasis, particularly to the liver (*p* = 0.002). Furthermore, the tumor grade of left-sided EOCC was generally better than that of right-sided EOCC (*p* < 0.001), but left-sided EOCC showed a higher incidence of perineural invasion. Notably, significant differences were observed in the therapeutic approaches employed for right-sided EOCC versus left-sided EOCC. These differences may contribute to the notable disparity in OS between the two groups (*p* = 0.002). Moreover, Kaplan–Meier survival analysis demonstrated that patients with right-sided EOCC exhibited worse OS and CSS compared to those with left-sided EOCC (Fig. 1A and Supplementary Fig. 1A).

**Table 1:** Characteristics of patients with EOCC in the right-side and left-side group

Characteristic	Right	Left	*P*-value
n	1980	3035	
Sex			< 0.001^*^
Female	907(45.8%)	1587(52.3%)	
Male	1073(54.2%)	1448(47.7)	
Histology			< 0.001^*^
Non-specific adenocarcinoma	1680(84.8%)	2847(93.8%)	
Specific adenocarcinoma	294(14.8%)	184(6.1%)	
Other	6(0.3%)	4(0.1%)	
Pathologic stage			0.002^*^
Stage I-II	807(40.8%)	1105 (36.4%)	
Stage III-IV	1173 (59.2%)	1930(63.6%)	
Surgery of Primary Site			0.006^*^
Yes	1965 (99.2%)	2985(98.4%)	
No	15(0.8%)	50(1.6%)	
Reginal lymph node dissection			< 0.001^*^
Yes	1937(97.8%)	2904 (95.7%)	
No	43(2.2%)	131 (4.3%)	
Radiation			0.006^*^
Yes	51(2.6%)	122(4.0%)	
No	1929(97.4)	2913(96.0%)	
Chemotherapy			< 0.001^*^
Yes	1217(61.5%)	2053(67.6%)	
No/unknown	763(38.5%)	982(32.4%)	
Bone metastasis			0.146
Yes	6(0.3%)	18(0.6%)	
No	1974(99.7%)	3017(99.4%)	
Brain metastasis			0.071
Yes	0(0%)	5 (0.2%)	
No	1980(100%)	3030(99.8%)	
Liver metastasis			0.002^*^
Yes	298(15.1%)	561(18.5%)	
No	1682(84.9%)	2474(81.5%)	
Lung metastasis			0.320
Yes	59(3.0%)	106 (3.5%)	
No	1921 (97.0%)	2929 (96.5%)	
Grade			< 0.001^*^
Well and moderate	1495(75.5%)	2564(84.5%)	
Poor	485(24.5%)	471(15.5%)	
Pretreatment CEA level			0.061
Negative	1190(60.1%)	1743(57.4%)	
Elevated	790(39.9%)	1292(42.6%)	
Perineural invasion			< 0.001^*^
Yes	305(15.4%)	603(19.9%)	
No	1675(84.6%)	2432(80.1%)	
Tumor size			< 0.001^*^
Median	55.00	45.00	
OS month			0.002^*^
Median	52.00	54.00	

^*^Statistical significance

In several previous studies, transverse-sided colon cancer was often grouped together with right-sided colon cancer [12]. However, due to the transverse colon’s position as the boundary between the midgut and hindgut, there is no definitive consensus in the literature regarding its origin. Therefore, it is important to explore potential differences between transverse-sided EOCC and right-sided EOCC. In our study, we compared 1980 patients with right-sided EOCC (35.4%) to 573 patients with transverse-sided EOCC (10.3%) (Table 2). Surprisingly, we found a significantly lower occurrence of regional lymph node metastasis (*p* = 0.048) and a higher proportion of patients in early stages for transverse-sided EOCC (*p* = 0.023). Additionally, transverse-sided EOCC demonstrated smaller tumor size (*p* < 0.001). Other clinical features, including OS, showed no significant differences between the two groups. Furthermore, our study revealed differences between transverse-sided EOCC and left-sided EOCC (Table 3). The differences observed between transverse-sided EOCC and left-sided EOCC mirrored those between right-sided EOCC and left-sided EOCC. However, the proportion of lung metastasis was significantly lower in transverse-sided EOCC (*p* = 0.030). Moreover, Kaplan–Meier survival analysis showed no significant difference in OS and CSS between transverse-sided EOCC and the other two groups (Fig. 1B, C and Supplementary Fig. 1B, C).

**Table 2:** Characteristics of patients with EOCC in the right-side and transverse-sided groups

Characteristic	Right	Transverse	*P*-value
n	1980	573	
Sex			0.855
Female	907(45.8%)	260(45.4%)	
Male	1073(54.2%)	313(54.6%)	
Histology			0.728
Non-specific adenocarcinoma	1680(84.8%)	484(84.5%)	
Specific adenocarcinoma	294(14.8%)	86(15.0%)	
Others	6(0.3%)	3(0.5%)	
Pathologic stage			0.048^*^
Stage I-II	807(40.8%)	260(45.4%)	
Stage III-IV	1173 (59.2%)	313(63.6%)	
Surgery of Primary Site			0.290
Yes	1965 (99.2%)	566(98.8%)	
No	15(0.8%)	7(1.2%)	
Reginal lymph node dissection			0.181
Yes	1937(97.8%)	555(96.9%)	
No	43(2.2%)	18(3.1%)	
Radiation, n (%)			0.006^*^
Yes	51(2.6%)	4(0.7%)	
No	1929(97.4)	569(99.3%)	
Chemotherapy			0.716
Yes	1217(61.5%)	357(62.3%)	
No/unknown	763(38.5%)	216(37.7%)	
Bone metastasis			0.182
Yes	6(0.3%)	4(0.7%)	
No	1974(99.7%)	569(99.3%)	
Liver metastasis			0.857
Yes	298(15.1%)	88(15.4%)	
No	1682(84.9%)	485(84.6%)	
Lung metastasis			0.109
Yes	59(3.0%%)	10(1.7%)	
No	1921 (97.0%)	563 (98.3%)	
Grade			0.250
Well and moderate	1495(75.5%)	446(77.8%)	
Poor	485(24.5%)	127(22.2%)	
Pretreatment CEA level			0.081
Negative	1190(60.1%)	321(56.0%)	
Elevated	790(39.9%)	252(44.0%)	
Perineural invasion			0.978
Yes	305(15.4%)	88(15.4%)	
No	1675(84.6%)	485(84.6%)	
Tumor size			< 0.001^*^
Median (mm)	55.00	50.00	
OS month			0.222
Median	52.00	54.00	

^*^Statistical significance

**Table 3:** Characteristics of patients with EOCC in the left-side and transverse-sided groups

Characteristic	Left	Transverse	*P*-value
n	3035	573	
Sex			0.002^*^
Female	1587(52.3%)	260 (45.4%)	
Male	1448(47.7%)	313 (54.6%)	
Histology			< 0.001^*^
Non-specific adenocarcinoma	2847 (93.8%)	484 (84.5%)	
Specific adenocarcinoma	184 (6.1%)	86 (15.0%)	
Others	4 (0.1%)	3 (0.5%)	
Pathologic stage			< 0.001^*^
Stage I-II	1105 (36.4%)	260 (45.4%)	
Stage III-IV	1930 (63.6%)	313 (63.6%)	
Surgery of Primary Site			0.453
Yes	2985 (98.4%)	566 (98.8%)	
No	50 (1.6%)	7 (1.2%)	
Reginal lymph node dissection			0.195
Yes	2904 (95.7%)	555 (96.9%)	
No	131 (4.3%)	18 (3.1%)	
Radiation			0.006^*^
Yes	122 (4.0%)	4 (0.7%)	
No	2913 (96.0%))	569 (99.3%)	
Chemotherapy			0.013^*^
Yes	2053(67.6%)	357 (62.3%)	
No/unknown	982 (32.4%)	216 (37.7%)	
Bone metastasis			0.767
Yes	18 (0.6%)	4 (0.7%)	
No	3017 (99.4%)	569 (99.3%)	
Liver metastasis			0.074^*^
Yes	561 (18.5%)	88 (15.4%)	
No	2474 (81.5%)	485 (84.6%)	
Brain metastasis			0.331
Yes	5 (0.2%)	0 (0%.0)	
No	3030 (99.8%)	573 (100.0%)	
Lung metastasis			0.030^*^
Yes	106 (3.5%)	10 (1.7%)	
No	2929 (96.5%)	563 (98.3%)	
Grade			< 0.001^*^
Well and moderate	2564 (84.5%)	446 (77.8%)	
Poor	471 (15.5%)	127 (22.2%)	
Pretreatment CEA level			0.532
Negative	1743 (57.4%)	321 (56.0%)	
Elevated	1292 (42.6%)	252 (44.0%)	
Characteristic	Left-sided	Transverse-sided	*P*-value
Perineural invasion			0.012^*^
Yes	603 (19.9%)	88 (15.4%)	
No	2432 (80.1%)	485 (84.6%)	
Tumor size			< 0.001^*^
Median (mm)	45.00	50.00	
OS month			0.458
Median	54	54	

^*^Statistical significance

To conduct further prognostic analysis, the patients with EOCC were randomly allocated to a training cohort and a validation cohort using a ratio of seven to three for right-sided EOCCs, left-sided EOCCs, and transverse-sided EOCCs, respectively. The patient baseline characteristics between the two cohorts were carefully balanced, ensuring comparability (Tables S1, S2 and S3). The training cohort was utilized for prognostic analysis and the construction of the nomogram, while the validation cohort was employed for internal validation. The results of univariate and multivariate analysis of OS and CSS using Cox regression analysis in the training cohort are presented in Tables 4, 5 and 6 and Supplementary Tables S7, S8 and S9.

**Table 4:** Univariate and multivariable Cox analysis for OS of the right-sided EOCCs

Characteristics	Univariate analysis	*P*-value	Multivariable analysis	*P*-value
	Hazard ratio (95% CI)		Hazard ratio (95% CI)	
Sex				
Female	Ref			
Male	0.857 (0.706–1.041)	0.120		
Histology				
Non-specific adenocarcinoma	Ref			
Specific adenocarcinoma	1.339 (1.029–1.743)	0.030^*^	0.676 (0.088–5.215)	0.707
Others	5.119 (0.934–28.039)	0.060		
Site				
Cecum	Ref			
Ascending colon	1.096 (0.802–1.498)	0.567		
Hepatic flexure	0.864 (0.628–1.186)	0.364		
Pathologic stage				
I-II	Ref			
III-IV	12.989 (9.493–17.772)	< 0.001^*^	7.350 (4.951–10.912)	< 0.001^*^
Surgery of Primary Site				
No	Ref			
Yes	0.029 (0.004–0.219)	0.013^*^	0.073 (0.005–1.124)	0.142
Reginal lymph node dissection				
No	Ref			
Yes	0.434 (0.280–0.671)	0.032^*^	1.084 (0.295–3.983)	0.903
Radiation				
No	Ref			
Yes	1.985 (1.335–2.952)	0.001^*^	1.849 (1.141–2.998)	0.005^*^
Chemotherapy				
No/unknown	Ref			
Yes	0.462 (0.368–0.582)	0.004^*^	0.516 (0.395–0.674)	0.002^*^
Bone metastasis				
No	Ref			
Yes	10.353 (0.876–23.176)	0.053		
Liver metastasis				
No	Ref			
Yes	1.508 (1.171–1.943)	0.001^*^	4.069 (3.211–5.157)	0.004^*^
Lung metastasis				
No	Ref			
Yes	1.633 (1.192–2.238)	0.002^*^	2.262 (1.583–3.231)	0.014^*^
Grade				
Poor	Ref			
Well and moderate	0.803 (0.733–0.878)	< 0.001^*^	0.508 (0.411–0.627)	0.004^*^
Pretreatment CEA				
Negative	Ref			
Elevated	1.402 (1.162–1.693)	< 0.001^*^	1.457 (1.168–1.817)	0.003^*^
Perineural invasion				
No	Ref			
Yes	1.736 (1.440–2.094)	< 0.001^*^	1.651 (1.324–2.059)	< 0.001^*^
Tumor size(mm)				
< 54.9	Ref			
> 54.9	1.347 (1.130–1.605)	0.001^*^	1.410 (1.079–1.842)	0.012^*^

^*^Statistical significance

**Table 5:** Univariate and multivariable Cox analysis for OS of the left-sided EOCCs

Characteristics	Univariate analysis	*P*-value	Multivariable analysis	*P*-value
	Hazard ratio (95% CI)		Hazard ratio (95% CI)	
Sex				
Female	Ref			
Male	1.264 (0.855–1.868)	0.178		
Histology				
Non-specific adenocarcinoma	Ref			
Specific adenocarcinoma	1.623 (0.795–2.087)	0.564		
Others	1.023 (0.587–1.673)	0.351		
Site				
Cecum	Ref			
Ascending colon	1.104 (0.758–1.606)	0.607		
Hepatic flexure	1.040 (0.799–1.352)	0.772		
Pathologic stage				
I-II	Ref			
III-IV	7.357 (5.762- 9.394)	< 0.001^*^	3.216 (2.332–4.436)	< 0.001^*^
Surgery of Primary Site				
No	Ref			
Yes	0.074 (0.036–0.152)	< 0.001^*^	0.297 (0.104–1.012)	0.203
Reginal lymph node dissection				
No	Ref			
Yes	0.373 (0.262–0.531)	0.021^*^	0.531 (0.284–1.193)	0.084
Radiation				
No	Ref			
Yes	1.305 (0.804–2.118)	0.282		
Chemotherapy				
No/unknown	Ref			
Yes	0.683 (0.502–0.916)	0.011^*^	0.738 (0.553–0.987)	0.040^*^
Bone metastasis				
No	Ref			
Yes	2.537 (0.531–12.122)	0.243		
Liver metastasis				
No	Ref			
Yes	13.236 (10.702–16.369)	< 0.001^*^	5.763 (4.517–7.352)	< 0.001^*^
Lung metastasis				
No	Ref			
Yes	9.599 (6.118–15.06)	< 0.001^*^	2.295 (1.354–3.890)	0.002^*^
Grade				
Well and moderate	Ref			
Poor	2.545 (2.075–3.122)	< 0.001^*^	2.222 (1.736–2.8450)	< 0.001^*^
Pretreatment CEA				
Negative	Ref			
Elevated	4.927 (4.130–5.878)	< 0.001^*^	2.148 (1.741–2.650)	< 0.001^*^
Perineural invasion				
No	Ref			
Yes	3.142 (2.605–3.789)	< 0.001^*^	1.777 (1.415–2.232)	< 0.001^*^
Tumor size (mm)				
< 44.9	Ref			
> 44.9	1.502 (1.275–1.769)	< 0.001^*^	1.067 (0.865–1.315)	0.547

^*^Statistical significance

**Table 6:** Univariate and multivariable Cox analysis for OS of the transverse-sided EOCCs

Characteristics	Univariate analysis	*P*-value	Multivariable analysis	*P*-value
	Hazard ratio (95% CI)		Hazard ratio (95% CI)	
Sex				
Female	Ref			
Male	0.815 (0.483–1.377)	0.445		
Histology				
Non-specific adenocarcinoma	Ref			
Specific adenocarcinoma	1.214 (0.657–3.965)	0.328		
Others	2.041 (0.186–4.813)	0.211		
Pathologic stage				
I-II	Ref			
III-IV	9.874 (5.884–16.569)	< 0.001^*^	4.789 (2.453–9.353)	< 0.001^*^
Surgery of Primary Site				
No	Ref			
Yes	2.593 (0.773–5.243)	0.487		
Reginal lymph node dissection				
No	Ref			
Yes	0.175 (0.065–0.475)	0.001^*^	0.167 (0.048–0.583)	0.005^*^
Radiation				
No	Ref			
Yes	2.848 (0.177–5.813)	0.460		
Chemotherapy				
No/unknown	Ref			
Yes	0.369 (0.186–0.734)	0.005^*^	0.526 (0.283–0.976)	0.042^*^
Bone metastasis				
No	Ref			
Yes	8.157 (0.842–79.012)	0.070		
Liver metastasis				
No	Ref			
Yes	14.196 (8.261–24.395)	< 0.001^*^	4.610 (2.445–8.691)	< 0.001^*^
Lung metastasis				
No	Ref			
Yes	11.216 (2.355–20.419)	0.002^*^	3.08 (0.571–16.616)	0.191
Grade				
Well and moderate	Ref			
Poor	2.985 (1.971–4.520)	< 0.001^*^	2.566 (1.517–4.342)	< 0.001^*^
Pretreatment CEA level				
Negative	Ref			
Elevated	6.558 (4.312–9.975)	< 0.001^*^	3.648 (2.216- 6.006)	< 0.001^*^
Perineural invasion				
No	Ref			
Yes	4.439 (2.768–7.117)	< 0.001^*^	2.710 (1.494- 4.919)	0.001^*^
Tumor size(mm)				
< 54.9	Ref			
> 54.9	0.391 (0.224–0.681)	0.001^*^	0.853 (0.590–1.233)	0.398

^*^Statistical significance

### Cox regression analysis for OS and CSS

In patients with right-sided EOCC, both univariate and multivariate Cox regression analyses were performed to determine the independent prognostic factors for OS and CSS. In the univariate analysis, several variables were found to have a significant impact on OS (Table 4), including histology, stage, primary site surgery, regional lymph node dissection, chemotherapy, radiation, liver metastases, lung metastases, grade, pretreatment CEA, perineural invasion, and tumor size which are also related to CSS (Supplementary Table S7).

After conducting the multivariate analysis, the following factors were identified as independent prognostic factors for OS in patients with right-sided EOCC: stage (HR: 7.350, 95% CI: 4.951–10.912), radiation (HR: 1.849, 95% CI: 1.141–2.998), chemotherapy (HR: 0.516, 95% CI: 0.395–0.674), liver metastasis (HR: 4.069, 95% CI: 3.211–5.157), lung metastasis (HR: 2.262, 95% CI: 1.583–3.231), grade (HR: 0.508, 95% CI: 0.411–0.627), elevated pretreatment CEA (HR: 1.457, 95% CI: 1.168–1.817), perineural invasion (HR: 1.651, 95% CI: 1.324–2.059), and bigger tumor size (HR: 1.410, 95% CI: 1.079–1.842) (Table 4). We also performed the multivariate analysis for CSS, which showed the same trends of the prognostic factors (Supplementary Table S7).

In patients with left-sided EOCC, univariate and multivariate Cox regression analyses were used to identify independent prognostic factors of OS and CSS. After univariate analysis, variables with a *P* < 0.05 including stage, primary site surgery, reginal lymph node dissection,chemotherapy, liver metastases, lung metastases, grade, pretreatment CEA, perineural invasion and tumor size were further investigated in multivariate Cox analysis. After multivariate analysis, stage ([stage III-IV vs stage I-II] HR: 3.216, 95%CI: 2.332–4.436); chemotherapy (HR:0.738, 95%CI: 0.553–0.987); liver metastasis (HR: 5.763, 95%CI: 4.517–7.352), lung metastasis (HR: 2.295, 95%CI:1.354–3.890); grade ([poor vs well and moderate] HR: 2.222, 95%CI:1.736–2.8450); elevated pretreatment CEA level ( HR: 2.148, 95%CI: 1.741–2.650); perineural invasion ( HR: 1.777, 95%CI: 1.415–2.232) (Table 5). Those prognostic factors performed the consistent results in univariate and multivariate analysis for CSS (Supplementary Table S8).

In the analysis of patients with transverse -sided EOCC, we conducted both univariate and multivariate Cox regression analyses to identify independent prognostic factors for OS and CSS. Variables with a significant association (*p* < 0.05) in the univariate analysis were further examined in the multivariate analysis. After thorough analysis, the following factors were identified as independent prognostic factors for OS in patients with transverse -sided EOCC: stage ([stage III-IV vs stage I-II] HR: 4.789, 95%CI: 2.453–9.353); reginal lymph node dissection (HR: 0.167,95%CI: 0.048–0.583); chemotherapy (HR: 0.526, 95%CI: 0.283–0.976); liver metastasis (HR: 4.610, 95%CI: 2.445–8.691); grade ([poor vs well and moderate] HR: 2.566, 95%CI: 1.517–4.342); elevated pretreatment CEA ( HR: 3.648, 95%CI: 2.216- 6.006); perineural invasion ( HR: 2.710, 95%CI: 1.494- 4.919) (Table 6). The univariate and multivariate analysis outcomes for CSS were almost consistent with the results of OS (Supplementary Table S9). What’s more, lung metastasis (HR: 2.598, 95%CI: 1.235–2.015) and bigger tumor size (HR: 1.578, 95%CI: 1.235–2.015) are identified as risk factors in CSS (Supplementary Table S9).

### Nomograms construction and validation

**Fig. 2:**
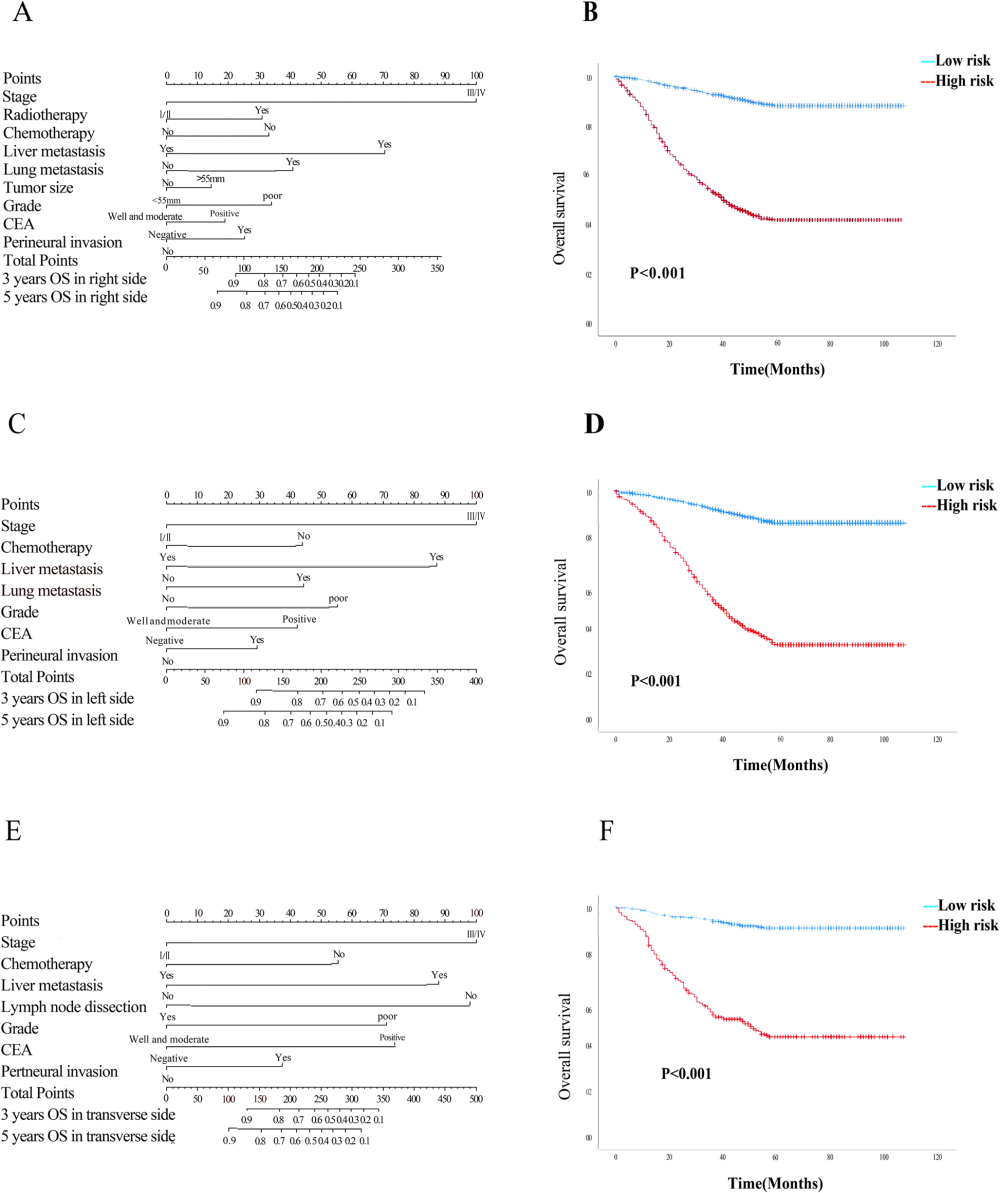
Nomograms for predicting the 3-year and 5-year OS rates of patients in right-sided EOCC, left-sided EOCC and transverse-sided EOCC (**A**, **C**, **E**). The Kaplan–Meier curves of the high-risk and low-risk groups in right-sided EOCC, left-sided EOCC and transverse-sided EOCC (**B**, **D**, **E**). Abbreviations: *EOCC* Early-onset colon cancer

In the training cohort, we constructed nomograms based on independent prognostic factors to predict the risk of OS and CSS at 3-year and 5-year intervals (Fig. 2A, C, E and Supplementary Fig. 2A, C, E). These nomograms provide a visual representation of the risk score for each patient, taking into account their individual characteristics. By employing X-tile software, we determined the optimal cut-off points for dividing patients into low-risk and high-risk groups for OS and CSS prediction. The cut-off points were found to be 200 (right-sided), 205 (left-sided), and 267.5 (transverse-sided) for the three tumor locations respectively in OS, which are so closed to the points in CSS. Remarkably, the survival outcomes between the low-risk and high-risk groups demonstrated significant differences for all three tumor locations (*p* < 0.001) (Fig. 2B, D, F and Supplementary Fig. 2B, D, F). Notably, patients in the low-risk group exhibited higher survival probabilities compared to those in the high-risk group.

**Fig. 3:**
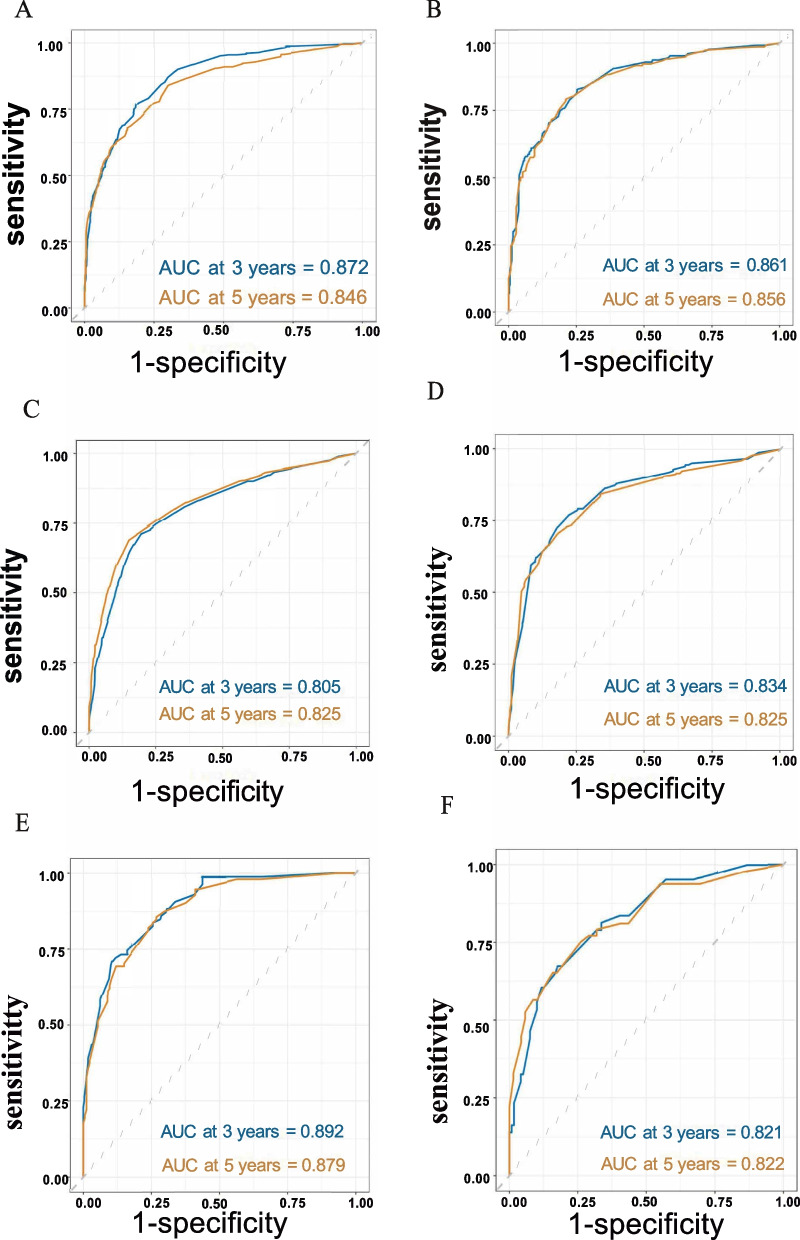
ROC curves of the nomogram for 3-year and 5-year OS in development cohort and validation cohort in right-sided EOCC (**A**, **B**), left-sided EOCC (**C**, **D**) and transverse-sided EOCC (**E**, **F**). Abbreviations: *EOCC* Early-onset colon cancer, *OS* overall survival

To evaluate the predictive accuracy of the nomograms, we calculated the area under the curve (AUC) values for OS and CSS probabilities at 3-year and 5-year timepoints. For the right-sided EOCC group, the AUC values in OS were 0.872 and 0.846 in the training cohort, and 0.861 and 0.856 in the validation cohort, respectively (Fig. 3A, B). Similarly, the nomograms demonstrated excellent predictive abilities for both 3-year and 5-year survival outcome in the left-sided EOCC and transverse-sided EOCC groups (Fig. 3C-F). Additionally, the performances of the AUC in CSS also showed excellent sensitivity and specificity for the predictive models (Supplementary Fig. 3). Discrimination ability of OS was assessed using the concordance index (C-index). The C-index for the right-sided EOCC group was 0.820 in the training cohort and 0.818 in the validation cohort. In the left-sided EOCC group, the C-index was 0.788 in the training cohort and 0.787 in the validation cohort. Moreover, for the transverse-sided EOCC group, the C-index was 0.845 in the training cohort and 0.835 in the validation cohort. The C-index of CSS had similar excellent outcomes. These values indicate a strong ability of the nomograms to discriminate between patients with different outcomes.

**Fig. 4:**
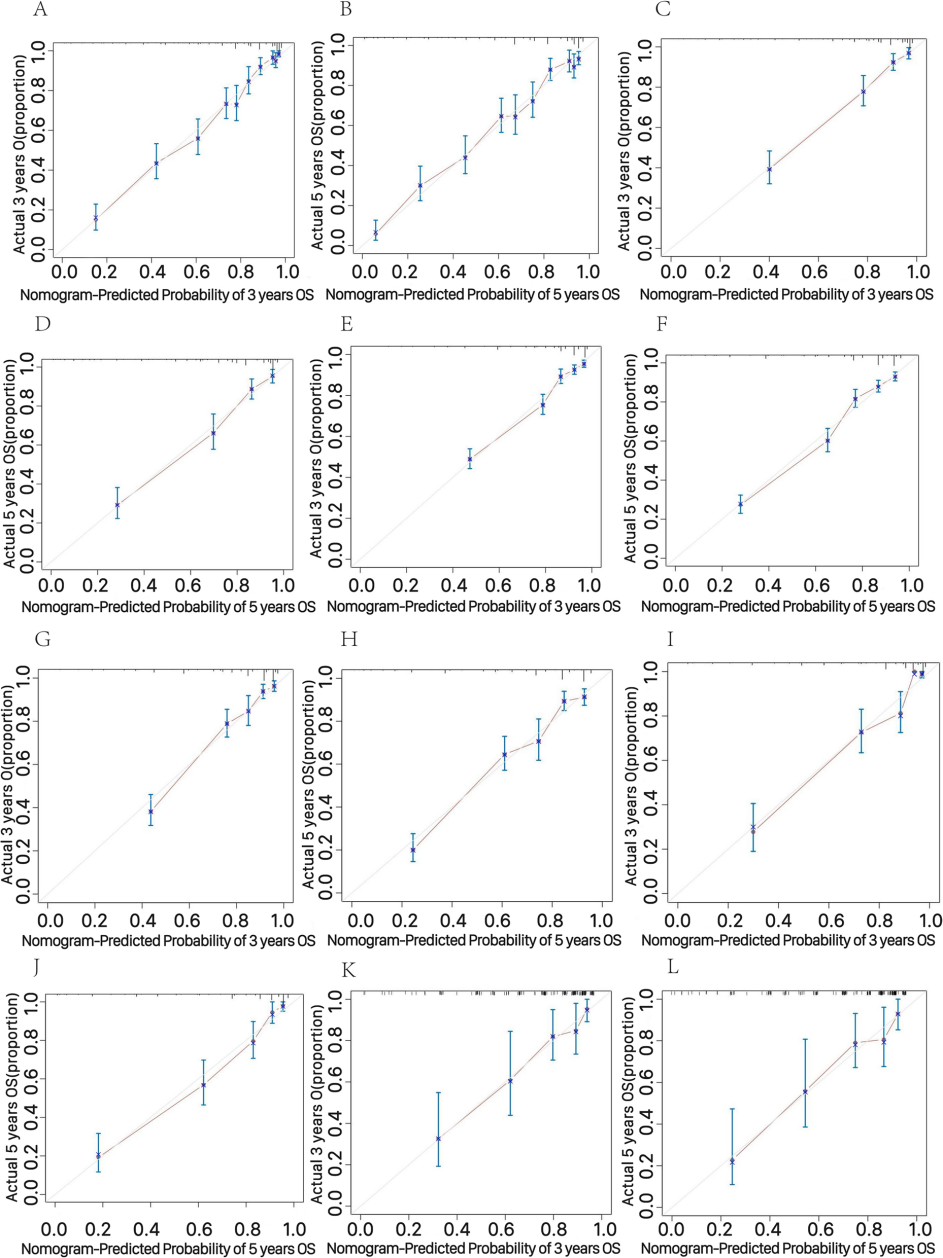
The calibration curves of the prognostic nomogram for 3-year and 5-year OS in the training sets and validation sets in right-sided EOCC (**A**-**D**), left-sided EOCC (**E**–**H**) and transverse-sided EOCC (**I**-**L**). Abbreviations: *EOCC* Early-onset colon cancer, *OS* overall survival

Calibration curves were plotted to evaluate the agreement between the predicted survival probabilities of OS and CSS by the nomograms and the observed survival probabilities at 3-year and 5-year timepoints for all three tumor groups (Fig. 4 and Supplementary Fig. 4). The calibration curves demonstrated good agreement, suggesting that the nomograms provide reliable estimations of survival probabilities.

**Fig. 5:**
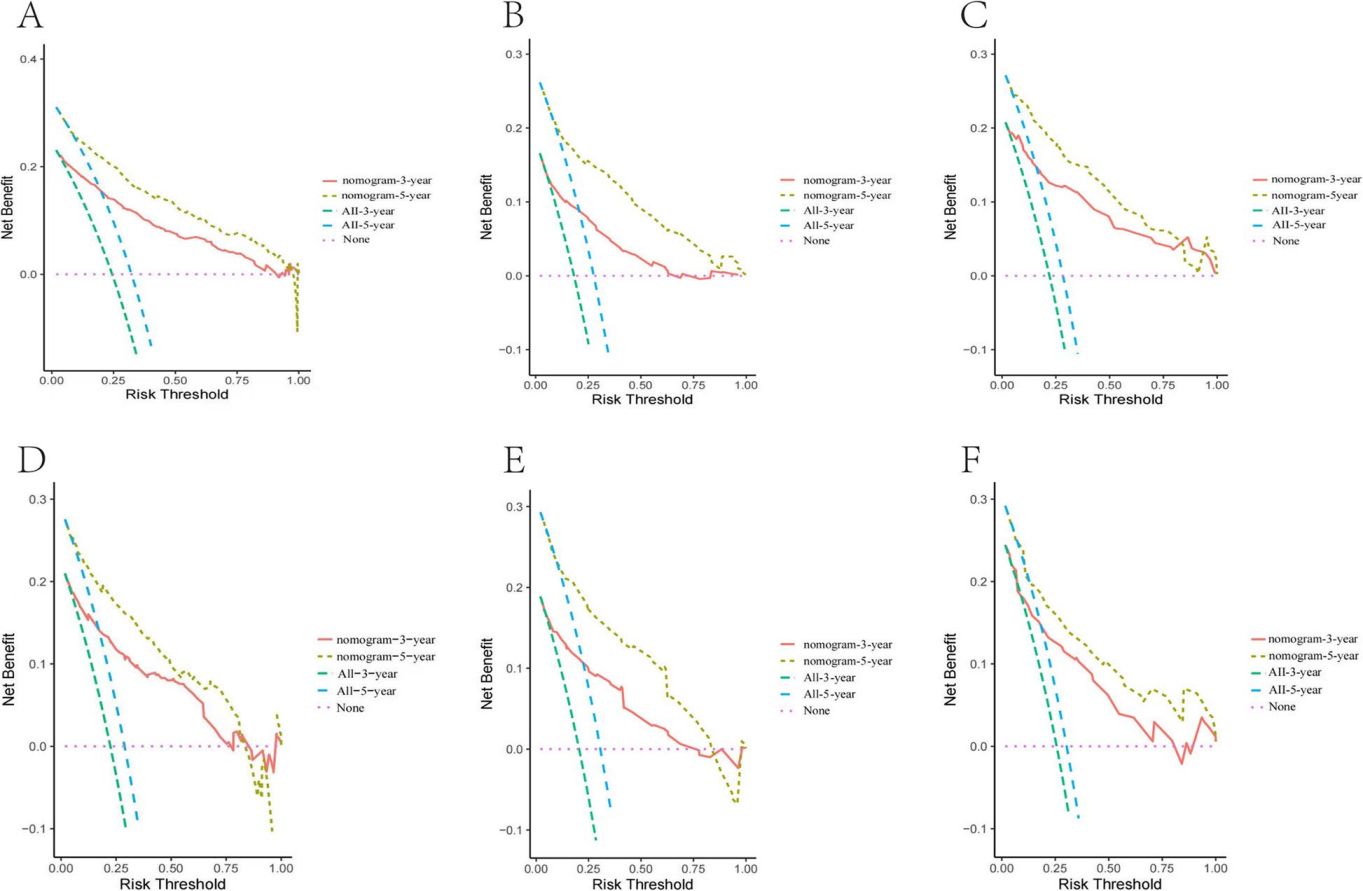
DCA of the OS nomogram models in the training sets and validation sets in right-sided EOCC (**A**, **B**), left-sided EOCC (**C**, **D**) and transverse-sided EOCC (**E**, **F**). Abbreviations: *DCA* Decision curve analysis, *EOCC* Early-onset colon cancer, *OS* overall survival

Furthermore, decision curve analysis (DCA) plots were performed to assess the clinical utility of the nomograms of OS and CSS. The DCA plots indicated that the nomograms have a threshold probability range of 0.1 to 0.9, within which they provide the better net benefit and higher clinical utility compared to either the treat-all strategy or the treat-none strategy. (Fig. 5A, C, E and Supplementary Fig. 5A, C, E). Importantly, these findings were consistent with the validation data (Fig. 5B, D, F and Supplementary Fig. 5B,D), further supporting the practical usefulness of the nomograms.

## Discussion

It is widely acknowledged that the incidence of EOCC has significantly increased over the past three decades [13]. Equally concerning is the fact that mortality rates from colorectal cancer (CRC) among adults younger than 50 years have been rising at a rate of 1.3% per year from 2008 to 2017, while mortality rates for individuals aged 65 years and older have been declining by 3% per year [14]. Moreover, the molecular characteristics of EOCC exhibit notable differences. As described by previous studies, early-onset CRC has a lower prevalence of somatic APC and BRAF mutations, but a higher prevalence of somatic CTNNB1 mutations when compared to late-onset CRC [14]. Additionally, in MSI-high tumors, BRAF mutations were found in 48% of older patients and only 5% of younger patients [15]. Not only age, but also tumor location plays a significant role in colon cancer. Right-sided and left-sided CRCs exhibit distinct underlying biological features, with a higher occurrence of MSI-high, CIMP-high, and BRAF mutant cancers observed among right-sided CRCs [16]. These characteristics are particularly prominent in the CMS1 genomic subtype, which is more prevalent in right-sided CRCs, although CMS3 also tends to favor right-sided CRCs. The differential distribution of these genomic CRC subtypes and other biological features between right-sided and left-sided CRCs may contribute to the inferior prognosis of advanced-stage right-sided CRCs and poorer outcomes with anti-EGFR therapy in right-sided CRC [17]. However, the precise definition of the boundary between right- and left-sided colon cancer is not universally standardized due to the embryological origin of the transverse colon from both the midgut and hindgut. In our study, we divided the colon into three segments and compared their clinical features separately. Our findings were consistent with previous research, demonstrating that left-sided EOCC had better survival outcomes compared to right-sided EOCC (54 months vs 52 months, *p* = 0.02) [18, 19]. Nevertheless, no significant difference was observed between the OS of transverse-sided EOCC and the OS of the other two locations.. Furthermore, our results indicated that left-sided EOCC patients had a higher proportion of advanced stage tumors (63.6% vs 59.2%, *p* = 0.002), but better tumor grade (84.5% vs 75.5%, *p* < 0.001) and smaller tumor size (45 mm vs 55 mm, *p* < 0.001) compared to right-sided EOCC patients, which aligns with previous studies [20, 21]. However, the presence of more perineural invasion (19.9% vs 15.4%, *p* < 0.001) and liver metastasis (18.5% vs 15.1%, *p* = 0.002) in left-sided EOCC patients contradicted earlier findings [22]. Few studies have compared the clinical features between transverse-sided EOCC and right-sided EOCC patients. In our study, we found that transverse-sided EOCC had a higher proportion of early-stage patients (45.4% vs 40.8%, *p* = 0.048) and smaller tumor size (50 mm vs 55 mm, *p* < 0.001), while other parameters showed no significant differences, suggesting that the tumor microenvironment in the transverse colon may be similar to that of the right colon.

The aim of our study was to develop simplified nomograms using multivariable regression analysis, which could more accurately assess the individual health of EOCC patients and predict their overall survival time, enabling the provision of personalized therapies [23]. Specifically, we constructed six distinct prognosis prediction models for EOCC patients’ OS and CSS based on the tumor’s location. In our study, we identified I several independent risk factors shared among all three groups, including advanced stage, liver metastasis, poor grade, pretreatment CEA level, and perineural invasion. Conversely, chemotherapy emerged as an independent protective factor, which aligns partly with previous research findings [24, 25]. Interestingly, our study revealed that radiotherapy was an independent risk factor in right-sided EOCC, which contradicts previous research indicating potential benefits of radiotherapy for CRC patients [26, 27].

Radiation therapy can be administered to target cancer cells either externally through a focused beam or via internally implanted radiation sources, with the goal of inducing cellular damage [28, 29]. Recent research has yielded promising results in the application of radiotherapy for CRC, including its combination with checkpoint blockade therapies and the use of stereotactic ablative radiotherapy for CRC with liver metastases [30, 31]. However, it’s worth noting that radiotherapy in rectal cancer may elevate the risk of bowel dysfunction and the development of radiotherapy resistance, which can result in treatment failure [32–34]. Moreover, when comparing the efficacy of surgery with or without adjuvant radiotherapy in T4 colon cancer, no statistically significant differences have been observed in terms of OS or disease-specific survival [35]. The potential factors contributing to our results are multifaceted. Firstly, patients who underwent radiotherapy exhibited a higher prevalence of advanced-stage disease (66.7% in stage III-IV) in contrast to those who didn’t receive radiotherapy (61.7% in stage III-IV). Secondly, it is imperative to acknowledge that the dataset pertaining to radiation therapy lacks completeness, potentially introducing a degree of bias into the evaluation of its therapeutic effects. Therefore, further high-level studies are required to determine the optimal use of radiotherapy in EOCC patients. Additionally, tumor size emerged as an independent risk factor in right-sided EOCC patients, although its prognostic value has been a subject of controversy in previous studies [9, 36]. In the context of colon cancer, the ideal extent of mesenteric resection and associated lymphadenectomy remains a topic of debate. Some research suggests that lymph node dissection of the gastrocolic ligament in patients with advanced proximal transverse-sided colon cancer may prolong survival time [37]. Intriguingly, in our study, we found that lymphadenectomy was an independent protective factor in transverse-sided EOCC patients, suggesting that extended lymphadenectomy may improve oncological outcomes in this particular group.

Some limitations remained in this study. Firstly, the SEER database lacks important biomarkers information like microsatellite instability (MSI), deficient mismatch repair (dMMR) which are vital prognostic factors [38]. In addition, the SEER database only provides basic therapy records and does not include supplementary details regarding specific surgical procedures, chemotherapy regimens, radiation doses, various health statuses, or socioeconomic factors that could potentially influence survival outcomes [39]. As a result, these valuable parameters could not be evaluated or integrated into our nomograms. Therefore, future studies should aim to incorporate these factors and assess their significance. Furthermore, due to the retrospective nature of this study, there is a possibility of selection bias among the enrolled patients. To validate our results and minimize bias, higher-level studies such as prospective cohort studies or randomized controlled trials are needed.

## Conclusion

This study highlights the significant impact of tumor location on the prognostic outcomes of patients with EOCC. It suggests that considering the tumor location is crucial for optimizing therapeutic strategies. Additionally, we successfully developed and validated nomograms for predicting OS and CSS in three specific tumor locations, using a large cohort of nearly 5500 EOCC patients. These nomograms offer a valuable solution to address the survival paradox observed with the AJCC staging system and serve as excellent tools for integrating clinical characteristics to guide therapeutic decision-making for EOCC patients. However, further prospective studies are needed to confirm and validate these findings.

### Supplementary Information

**Additional file 1: Supplementary Fig. 1.** The Kaplan-Meier CSS analysis in three different tumor locations.

**Additional file 2: Supplementary Fig. 2.** Nomograms for predicting the 3-year and 5-year CSS rates of patients in right-sided EOCC, left-sided EOCC and transverse-sided EOCC (A,C,E).

**Additional file 3: Supplementary Fig. 3.** ROC curves of the nomogram for 3-year and 5-year CSS in development cohort and validation cohort in right-sided EOCC (A-B), left-sided EOCC (C-D) and transverse-sided EOCC (E-F).

**Additional file 4: Supplementary Fig. 4.** The calibration curves of the prognostic nomogram for 3-year and 5-year CSS in the training sets and validation sets in right-sided EOCC (A-D), left-sided EOCC (E-H) and transverse-sided EOCC (I-L).

**Additional file 5: Supplementary Fig. 5.** DCA of the CSS nomogram models in the training sets and validation sets in right-sided EOCC (A-B), left-sided EOCC (C-D) and transverse-sided EOCC (E-F).

**Additional file 6: Table S1.** Baseline characteristics of right-sided EOCC patients in the training and validation cohorts

**Additional file 7: Table S2.** Baseline characteristics of the left-sided EOCC patients in the training and validation cohorts.

**Additional file 8: Table S3.** Baseline characteristics of the transversed-sided EOCC patients in the training and validation cohorts.

**Additional file 9: Table S4.** Baseline characteristics of right-sided EOCC patients in the training and validation cohorts for CSS.

**Additional file 10: Table S5.** Baseline characteristics of left-sided EOCC patients in the training and validation cohorts for CSS.

**Additional file 11: Table S6.** Baseline characteristics of the transversed-sided EOCC patients in the training and validation cohorts for CSS.

**Additional file 12: Table S7.** Univariate and multivariable Cox analysis for CSS of the right-sided EOCCs.

**Additional file 13: Table S8.** Univariate and multivariable Cox analysis for CSS of the left-sided EOCCs.

**Additional file 14: Table S9.** Univariate and multivariable Cox analysis for OS of the transverse-sided EOCCs.

## Data Availability

The datasets acquired and assessed in the course of this research are archived within the SEER database, accessible at https://seer.cancer.gov/. For inquiries regarding study data, Qiang Feng can be contacted through a reasonable request.

## Availability of data and materials

The original contributions presented in the study are included in the article/supplementary material. Further inquiries can be directed to the corresponding author.
