# Supplementary material for: Development and validation of prognostic nomograms for early-onset colon cancer in different tumor locations: a population-based study
Source: BMC Gastroenterol. 2023 Oct 21;23:362. doi: 10.1186/s12876-023-02991-1 (PMC10590526; doi:10.1186/s12876-023-02991-1)
Supplement: Supplementary file 1 — Additional file 1: Supplementary Fig. 1. The Kaplan-Meier CSS analysis in three different tumor locations. [file 12876_2023_2991_MOESM1_ESM.pdf]

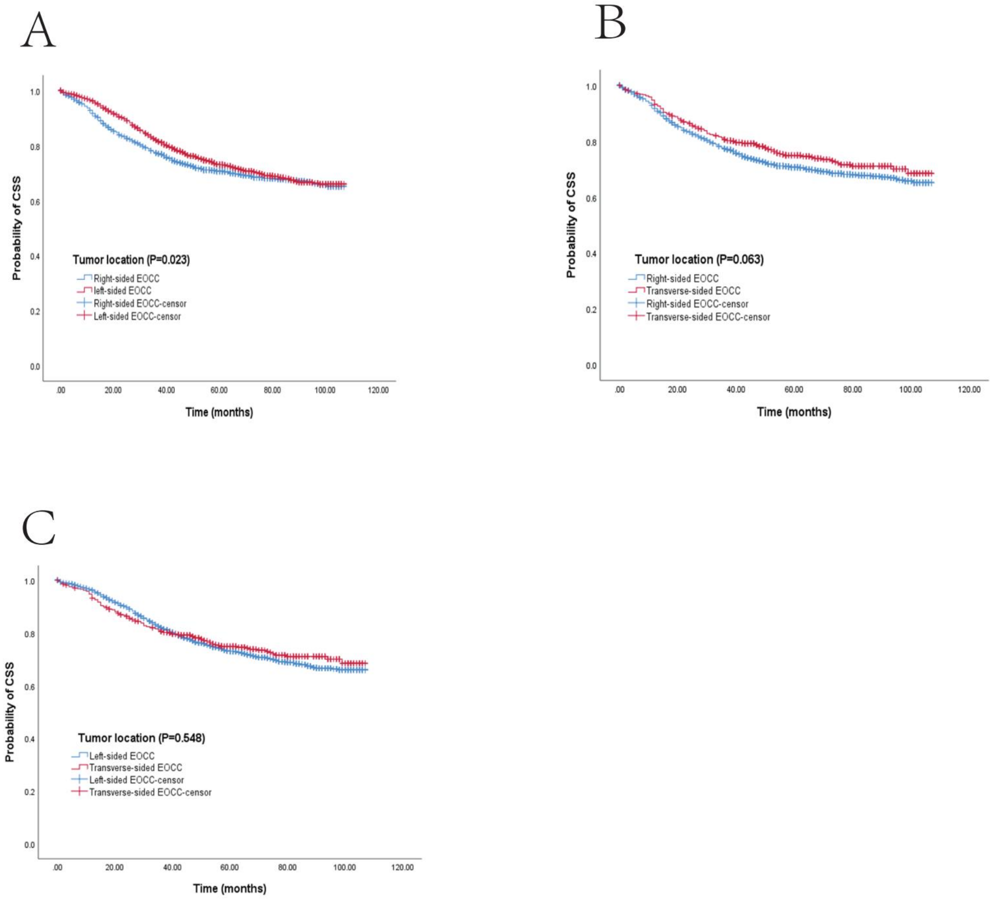


Supplementary Fig.1 The Kaplan-Meier CSS analysis in three different tumor locations.(A) The Kaplan-Meier survival analysis between right-sided EOCCs and left-sided EOCCs.(B) The Kaplan-Meier survival analysis between right-sided EOCCs and transverse-sided EOCCs.(C) The Kaplan-Meier survival analysis between transverse-sided EOCCS and left-sided EOCCs. Abbreviations: *CSS* Cancer-specific survival ; *EOCC* Early-onset colon cancer.
