# Supplementary material for: Development and validation of prognostic nomograms for early-onset colon cancer in different tumor locations: a population-based study
Source: BMC Gastroenterol. 2023 Oct 21;23:362. doi: 10.1186/s12876-023-02991-1 (PMC10590526; doi:10.1186/s12876-023-02991-1)
Supplement: Supplementary file 2 — Additional file 2: Supplementary Fig. 2. Nomograms for predicting the 3-year and 5-year CSS rates of patients in right-sided EOCC, left-sided EOCC and transverse-sided EOCC (A,C,E). [file 12876_2023_2991_MOESM2_ESM.pdf]

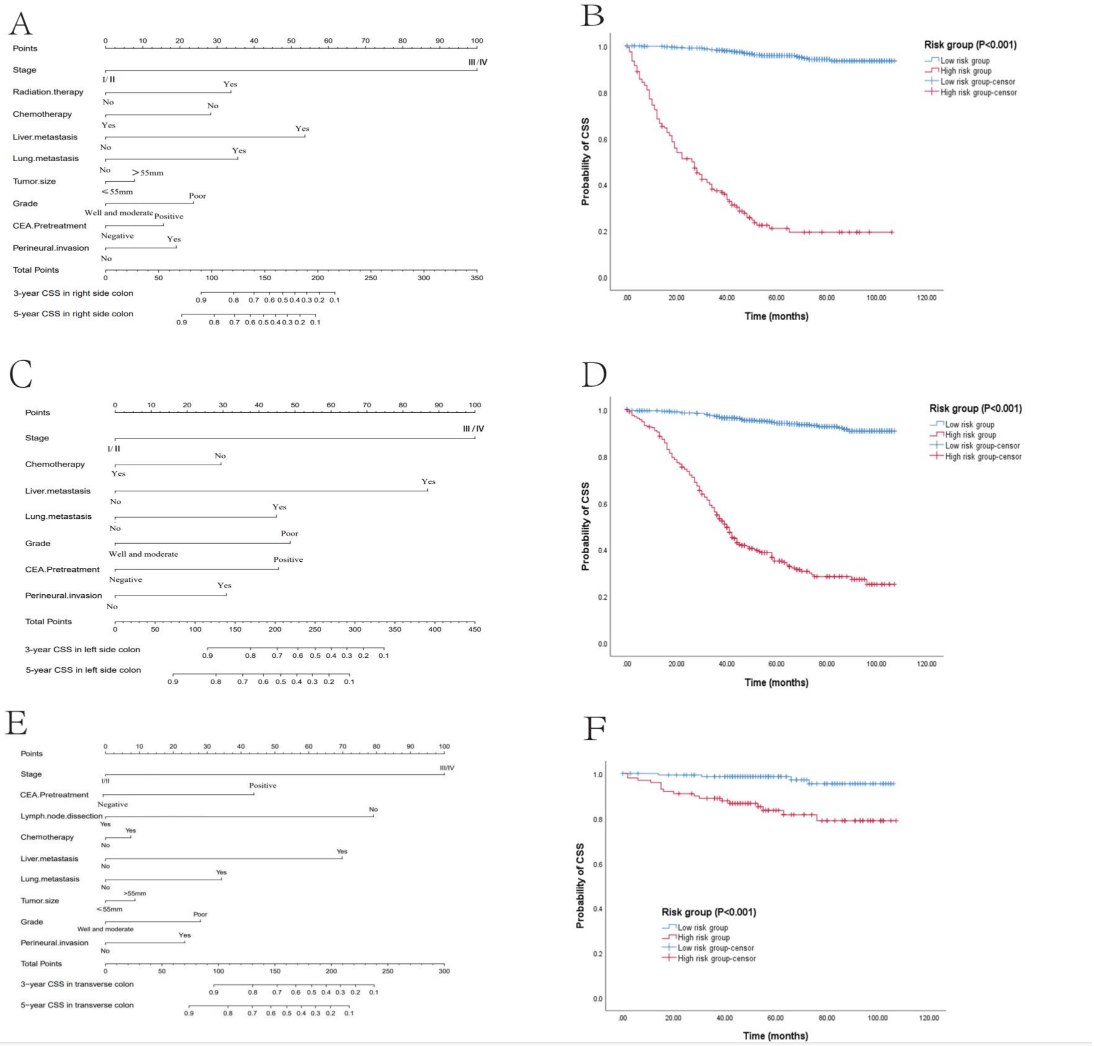


Supplementary Fig.2 Nomograms for predicting the 3-year and 5-year CSS rates of patients in right-sided EOCC, left-sided EOCC and transverse-sided EOCC (A,C,E). The Kaplan-Meier curves of the high-risk and low-risk groups in right-sided EOCC, left-sided EOCC and transverse-sided EOCC (B,D,F). Abbreviations: *CSS* Cancer-specific survival ;*EOCC* Early-onset colon cancer.
