# Supplementary material for: Development and validation of prognostic nomograms for early-onset colon cancer in different tumor locations: a population-based study
Source: BMC Gastroenterol. 2023 Oct 21;23:362. doi: 10.1186/s12876-023-02991-1 (PMC10590526; doi:10.1186/s12876-023-02991-1)
Supplement: Supplementary file 3 — Additional file 3: Supplementary Fig. 3. ROC curves of the nomogram for 3-year and 5-year CSS in development cohort and validation cohort in right-sided EOCC (A-B), left-sided EOCC (C-D) and transverse-sided EOCC (E-F). [file 12876_2023_2991_MOESM3_ESM.pdf]

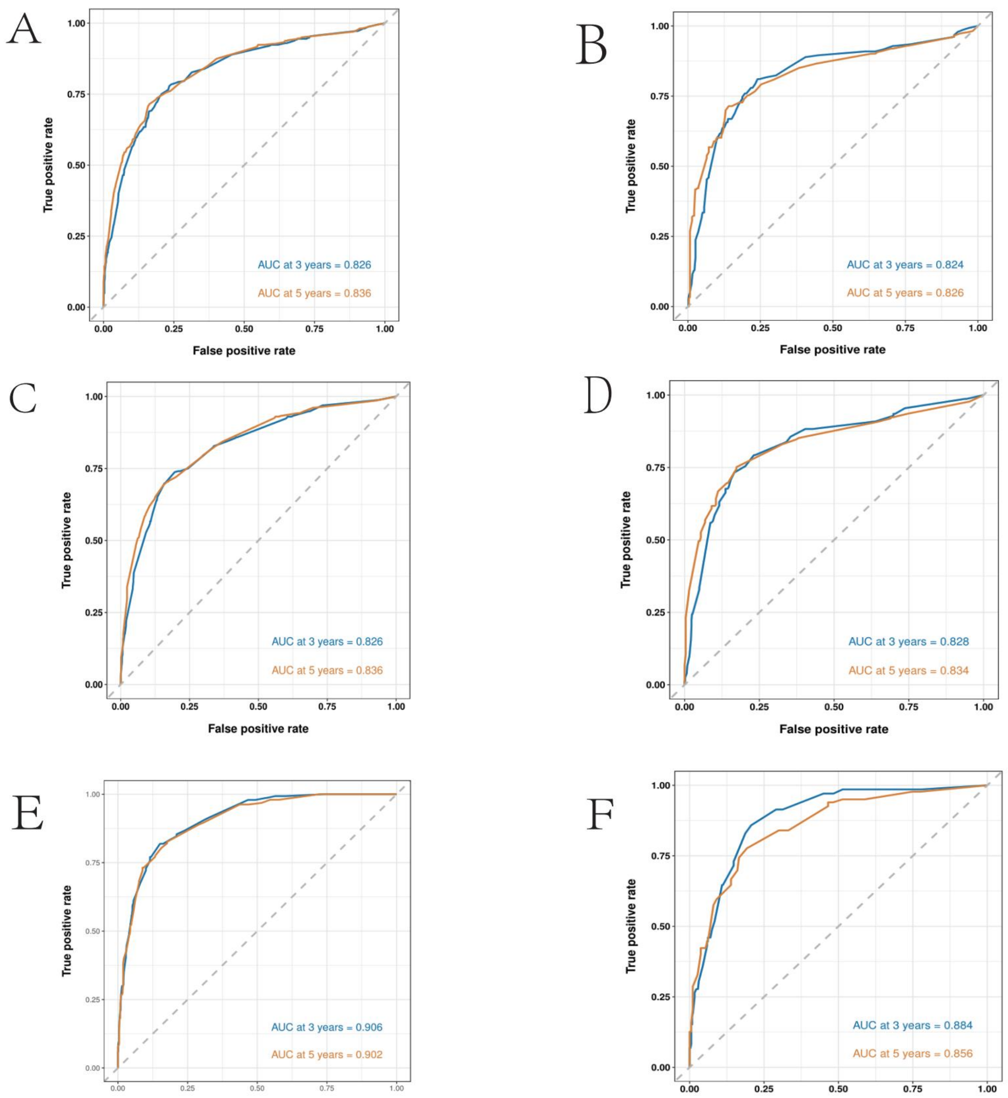

Supplementary Fig.3 ROC curves of the nomogram for 3-year and 5-year CSS in development cohort and validation cohort in right-sided EOCC (A-B), left-sided EOCC (C-D) and transverse-sided EOCC (E-F). Abbreviations: *EOCC* Early-onset colon cancer, *CSS* Cancer-specific survival.
