# Supplementary material for: Development and validation of prognostic nomograms for early-onset colon cancer in different tumor locations: a population-based study
Source: BMC Gastroenterol. 2023 Oct 21;23:362. doi: 10.1186/s12876-023-02991-1 (PMC10590526; doi:10.1186/s12876-023-02991-1)
Supplement: Supplementary file 6 — Additional file 6: Table S1. Baseline characteristics of right-sided EOCC patients in the training and validation cohorts [file 12876_2023_2991_MOESM6_ESM.docx]

| Table S1 Baseline characteristics of right-sided EOCC patients in the training and validation cohorts | | | | |
| --- | --- | --- | --- | --- |
| Characteristic | All cohort  n=1980  *N*(%) | Training cohort  n=1386  N(%) | Validation cohort  n=594  *N*(%) | *P*-value |
| sex |  |  |  | 0.454 |
| Female | 907 | 643 (46.4%) | 264 (44.4%) |  |
| Male | 1073 | 743 (53.6%) | 330 (55.6%) |  |
| Histology |  |  |  | 0.708 |
| Non-specific adenocarcinoma | 1680 | 1171 (84.5%) | 509 (85.7%) |  |
| specific adenocarcinoma | 294 | 210 (15.2%) | 84 (14.1%) |  |
| other | 6 | 5 (0.4%) | 1 (0.2%) |  |
| Site |  |  |  | 0.733 |
| cecum | 896 | 623 (44.9%) | 273 (46%) |  |
| ascending Colon | 847 | 592 (42.7%) | 255 (42.9%) |  |
| Hepatic Flexure | 237 | 171 (12.3%) | 66 (11.1%) |  |
| T stage, n (%) |  |  |  | 0.374 |
| T1-2 | 326 | 221 (15.9%) | 105 (17.7%) |  |
| T3-4 | 1654 | 1165 (84.1%) | 489 (82.3%) |  |
| N stage |  |  |  | 0.828 |
| N0 | 861 | 600 (43.3%) | 261 (43.9%) |  |
| N1-2 | 1119 | 786 (56.7%) | 333 (56.1%) |  |
| M stage |  |  |  | 0.983 |
| M0 | 1549 | 1084 (78.2%) | 465 (78.3%) |  |
| M1 | 431 | 302 (21.8%) | 129 (21.7%) |  |
| Pathologic stage |  |  |  | 0.964 |
| Stage I-II | 807 | 565 (40.8%) | 242 (40.7%) |  |
| Stage III-IV | 1173 | 821 (59.2%) | 352 (59.3%) |  |
| Surgery of Primary Site |  |  |  | 0.781 |
| Yes | 1965 | 1376 (99.3%) | 589 (99.2%) |  |
| No | 15 | 10 (0.7%) | 5 (0.8%) |  |
| Reginal lymph node dissection |  |  |  | 0.893 |
| Yse | 1937 | 1355 (97.8%) | 582 (98.0%) |  |
| No | 43 | 31 (2.2%) | 12 (2.0%) |  |
| Characteristic | All cohort  n=1980  N(%) | Training cohort  n=1386  N(%) | Validation cohort  n=594  N(%) | *P-*value |
| Radiation, |  |  |  | 0.496 |
| Yes | 51 | 33 (2.4%) | 18 (3%) |  |
| No | 1929 | 1353 (97.6%) | 576 (97%) |  |
| Chemotherapy |  |  |  | 0.717 |
| Yes | 1217 | 856 (61.8%) | 361 (60.8%) |  |
| No/unknown | 763 | 530 (38.2%) | 233 (39.2%) |  |
| Bone metastasis |  |  |  | 0.373 |
| Yes | 6 | 3 (0.2%) | 3 (0.5%) |  |
| No | 1974 | 1383 (99.8%) | 591 (99.5%) |  |
| Liver mestasis |  |  |  | 0.773 |
| Yes | 298 | 206 (14.9%) | 92 (15.5%) |  |
| No | 1882 | 1180 (85.1%) | 502 (84.5%) |  |
| Lung mestasis |  |  |  | 0.356 |
| Yes | 59 | 45 (3.2%) | 14 (2.4%) |  |
| No | 1921 | 1341 (96.8%) | 580 (97.6%) |  |
| Grade, n (%) |  |  |  | 0.937 |
| Well and moderate | 1495 | 1046 (75.5%) | 449 (75.6%) |  |
| poor | 485 | 340(24.5%) | 145(24.4%) |  |
| Pretreatment CEA level |  |  |  | 0.582 |
| negative | 1190 | 839 (60.5%) | 351 (59.1%) |  |
| elevated | 790 | 547 (39.5%) | 243 (40.9%) |  |
| Perineural invasion |  |  |  | 0.984 |
| Yse | 305 | 214 (15.4%) | 91 (15.3%) |  |
| No | 1675 | 1172 (84.6%) | 503 (84.7%) |  |
| Tumor size(mm) |  |  |  | 0.876 |
| <54.9 | 908 | 641(46.2%) | 267(44.9% |  |
| >54.9 | 1072 | 745(53.8%) | 327(54.1%) |  |
| Survival status |  |  |  | 0.798 |
| Alive | 1403 | 987(71.2%) | 416（70.0%） |  |
| Dead | 577 | 399(29.9) | 178（30.0%） |  |
