# Supplementary material for: Development and validation of prognostic nomograms for early-onset colon cancer in different tumor locations: a population-based study
Source: BMC Gastroenterol. 2023 Oct 21;23:362. doi: 10.1186/s12876-023-02991-1 (PMC10590526; doi:10.1186/s12876-023-02991-1)
Supplement: Supplementary file 7 — Additional file 7: Table S2. Baseline characteristics of the left-sided EOCC patients in the training and validation cohorts. [file 12876_2023_2991_MOESM7_ESM.docx]

| Table S2 Baseline characteristics of the left-sided EOCC patients in the training and validation cohorts | | | | |
| --- | --- | --- | --- | --- |
| Characteristic | All cohort  n=3035  *N*(%) | Training cohort  n=2125  N(%) | Validation cohort  n=910  *N*(%) | *P-* value |
| sex |  |  |  | 0.712 |
| Female | 1587（52.3%） | 1106 (52%) | 481 (52.9%) |  |
| Male | 1448（47.7%） | 1019 (48%) | 429 (47.1%) |  |
| Histology |  |  |  | 0.879 |
| Non-specific adenocarcinoma | 2847（93.8%） | 1993(93.76%) | 854 (93.89%) |  |
| specific adenocarcinoma | 184（6.0%） | 129 (6.1%) | 55 (6.0%) |  |
| other | 4（0.2%） | 3(0.14%) | 1 (0.11%) |  |
| Site |  |  |  | 0.641 |
| Splenic Flexure | 230（7.6%） | 155 (7.3%) | 75 (8.2%) |  |
| Descending Colon | 569（18.7%） | 397 (18.7%) | 172 (18.9%) |  |
| Sigmoid Colon | 2236（73.7%） | 1573 (74.0%) | 663 (72.9%) |  |
| T stage, n (%) |  |  |  | 0.359 |
| T1-2 | 596（19.6%） | 427 (20.1%) | 169 (18.6%) |  |
| T3-4 | 2439（80.4%） | 1698 (79.9%) | 741 (81.4%) |  |
| N stage |  |  |  | 0.601 |
| N0 | 1244（41.0%） | 878 (41.3%) | 366 (40.2%) |  |
| N1-2 | 1791（59.0%） | 1247 (58.7%) | 544 (59.8%) |  |
| M stage |  |  |  | 0.606 |
| M0 | 2285（75.3%） | 1606 (75.6%) | 679 (74.6%) |  |
| M1 | 750（24.7） | 519 (24.4%) | 231 (25.4%) |  |
| Pathologic stage |  |  |  | 0.964 |
| Stage I-II | 1105（36.4%） | 779 (36.7%) | 326 (35.8%) |  |
| Stage III-IV | 1930（63.6%） | 1346 (63.3%) | 584 (64.2%) |  |
| Surgery of Primary Site |  |  |  | 0.087 |
| Yes | 2985（98.4%） | 2084 (98.1%) | 901 (99%) |  |
| No | 50（1.6%） | 41 (1.9%) | 9 (1%) |  |
| Reginal lymph node dissection |  |  |  | 0.260 |
| Yse | 2904（95.7%） | 2027 (95.4%) | 877 (96.4%) |  |
| No | 131（4.3%） | 98 (4.6%) | 33 (3.6%) |  |
|  |  |  |  |  |
|  |  |  |  |  |
| Characteristic | All cohort  n=3035  N(%) | Training cohort  n=2125  N(%) | Validation cohort  n=910  N(%) | *P*-value |
| Radiation |  |  |  | 0.206 |
| Yes | 122 (4.0%) | 84 (4.0%) | 38 (4.2%) |  |
| No | 2913 (96.0%) | 2041 (96.0%) | 872 (95.8%) |  |
| Chemotherapy |  |  |  | 0.717 |
| Yes | 2053（67.6%） | 1422 (66.9%) | 631 (69.3%) |  |
| No/unknown | 982（32.4%） | 703 (33.1%) | 279 (30.7%) |  |
| Bone metastasis |  |  |  | 0.985 |
| Yes | 18 (0.6%) | 13 (0.6%) | 5 (0.5%) |  |
| No | 3017 (99.4%) | 2112 (99.4%) | 905 (99.5%) |  |
| Liver metastasis |  |  |  | 0.249 |
| Yes | 561 (18.5%) | 381 (17.9%) | 180 (19.8%) |  |
| No | 2474 (81.5%) | 1744 (82.1%) | 730 (80.2%) |  |
| Lung metastasis |  |  |  | 0.422 |
| Yes | 106 (3.5%) | 70 (3.3%) | 36 (4.0%) |  |
| No | 2929 (96.5%) | 2055 (96.7%) | 874 (96.0%) |  |
| Brain metastasis |  |  |  | 0.331 |
| Yes | 5 (0.2%) | 4 (0.2%) | 1 (0.1%) |  |
| No | 3030 (99.8%) | 2121 (99.8) | 909 (99.9%) |  |
| Grade, n (%) |  |  |  | 0.976 |
| Well and moderate | 2564 (84.5%) | 1796 (84.5%) | 768 (84.4%) |  |
| poor | 471 (15.5%) | 329 (15.5%) | 142 (15.6%) |  |
| Pretreatment CEA level |  |  |  | 0.147 |
| negative | 1743 (57.4%) | 1239 (58.3%) | 504 (55.4%) |  |
| elevated | 1292（42.5%） | 886 (41.7%) | 406 (44.6%) |  |
| Perineural invasion |  |  |  | 0.288 |
| Yse | 603（19.9%） | 411 (19.3%) | 192 (21.1%) |  |
| No | 2432（80.1%） | 1714 (80.7%) | 718 (78.9%) |  |
| Tumor size(mm) |  |  |  | 0.748 |
| <44.9 | 1516（49.9%） | 1066 (50.2%) | 450 (49.5%) |  |
| >44.9 | 1519（50.1%） | 1059 (49.8%) | 460 (50.5%) |  |
| Survival status |  |  |  | 0.798 |
| Alive | 2244（46.2%） | 1574(74.1%) | 670（73.6%） |  |
| Dead | 1632（53.8%） | 554 (25.9%) | 240（26.4%） |  |
