# Supplementary material for: Development and validation of prognostic nomograms for early-onset colon cancer in different tumor locations: a population-based study
Source: BMC Gastroenterol. 2023 Oct 21;23:362. doi: 10.1186/s12876-023-02991-1 (PMC10590526; doi:10.1186/s12876-023-02991-1)
Supplement: Supplementary file 8 — Additional file 8: Table S3. Baseline characteristics of the transversed-sided EOCC patients in the training and validation cohorts. [file 12876_2023_2991_MOESM8_ESM.docx]

| Table S3 Baseline characteristics of the transversed-sided EOCC patients in the training and validation cohorts | | | | |
| --- | --- | --- | --- | --- |
| Characteristic | All cohort  n=573  *N*(%) | Training cohort  n=402  N(%) | Validation cohort  n=171  *N*(%) | *P*-value |
| sex |  |  |  | 0.571 |
| Female | 260（45.4%） | 186 (46.3%) | 74 (43.3%) |  |
| Male | 313（54.6%） | 216 (53.7%) | 97 (56.7%) |  |
| Histology |  |  |  | 0.121 |
| Non-specific adenocarcinoma | 484（84.5%） | 332 (82.6%) | 152 (88.9%) |  |
| specific adenocarcinoma | 86（15.0%） | 68 (16.9%) | 18 (10.5%) |  |
| other | 3（0.5%） | 2(0.5%) | 1 (0.6%) |  |
| T stage, n (%) |  |  |  | 0.415 |
| T1-2 | 79（13.8%） | 59 (14.7%) | 20 (11.7%) |  |
| T3-4 | 494（86.2%） | 343 (85.3%) | 151 (88.3%) |  |
| N stage |  |  |  | 0.893 |
| N0 | 277（48.3%） | 190(47.3%) | 87 (50.9%) |  |
| N1-2 | 296（51.7%） | 212(52.7%) | 84 (49.1%) |  |
| M stage |  |  |  | 0.274 |
| M0 | 444（77.5%） | 317 (78.9%) | 127 (74.3%) |  |
| M1 | 129（22.5%）） | 85 (21.1%) | 44 (25.7%) |  |
| Pathologic stage |  |  |  | 0.453 |
| Stage I-II | 260（45.4%） | 187 (46.5%) | 73 (42.7%) |  |
| Stage III-IV | 313（54.6%） | 215 (53.5%) | 98 (57.3%) |  |
| Surgery of Primary Site |  |  |  | 0.680 |
| Yes | 566（98.8%） | 396 (98.5%) | 170 (99.4%) |  |
| No | 7（1.2%） | 6 (1.5%) | 1 (0.6%) |  |
| Reginal lymph node dissection |  |  |  | 0.648 |
| Yse | 555（96.9%） | 388 (96.5%) | 167 (97.7%) |  |
| No | 18（3.1%） | 14 (3.5%) | 4 (2.3%) |  |
|  |  |  |  |  |
|  |  |  |  |  |
| Characteristic | All cohort  n=573  N(%) | Training cohort  n=402  N(%) | Validation cohort  n=171  N(%) | *P*-value |
| Radiation |  |  |  | 0.323 |
| Yes | 4（0.7%） | 3 (0.7%) | 1（0.6%） |  |
| No | 569 (99.3%) | 398 (99.3%) | 170 (99.4%) |  |
| Chemotherapy |  |  |  | 0.456 |
| Yes | 357（62.3%） | 246 (61.2%) | 111 (64.9%) |  |
| No/unknown | 216（37.7%） | 156 (38.8%) | 60 (35.1%) |  |
| Bone metastasis |  |  |  | 0.587 |
| Yes | 4 (0.7%) | 2 (0.5%) | 2 (1.2%) |  |
| No | 569（99.3） | 400 (99.5%) | 169 (98.8%) |  |
| Liver metastasis |  |  |  | 0.571 |
| Yes | 88 (15.4%) | 59 (14.7%) | 29 (17%) |  |
| No | 485 (84.6%) | 343 (85.3%) | 142 (83%) |  |
| Lung metastasis |  |  |  | 0.422 |
| Yes | 10 (1.7%) | 6 (1.5%) | 4 (2.3%) |  |
| No | 563 (98.3%) | 396 (98.5%) | 167 (97.7%) |  |
| Grade, n (%) |  |  |  | 0.598 |
| Well and moderate | 446 (77.8%) | 310 (77.1%) | 136 (79.5%) |  |
| poor | 127 (22.2%) | 92 (22.9%) | 35 (20.5%) |  |
| Pretreatment CEA level |  |  |  | 0.923 |
| negative | 321 (56.0%) | 225 (56.0%) | 96 (56.1%) |  |
| elevated | 252 (44.0%） | 177 (44.0%) | 75 (43.9%) |  |
| Perineural invasion |  |  |  | 0.952 |
| Yse | 88 (15.4%） | 61 (15.2%) | 27 (15.8%) |  |
| No | 485 (84.5%） | 341 (84.8%) | 144 (84.2%) |  |
| Tumor size (mm) |  |  |  | 0.667 |
| <49.9 | 292（51.0%） | 202 (50.2%) | 90 (52.6%) |  |
| >49.9 | 281（49.0%） | 200 (49.8%) | 81 (47.4%) |  |
| OS month |  |  |  | 0.964 |
| median | 54 | 54 | 54 |  |
