# Supplementary material for: Development and validation of prognostic nomograms for early-onset colon cancer in different tumor locations: a population-based study
Source: BMC Gastroenterol. 2023 Oct 21;23:362. doi: 10.1186/s12876-023-02991-1 (PMC10590526; doi:10.1186/s12876-023-02991-1)
Supplement: Supplementary file 9 — Additional file 9: Table S4. Baseline characteristics of right-sided EOCC patients in the training and validation cohorts for CSS. [file 12876_2023_2991_MOESM9_ESM.docx]

| Table S4 Baseline characteristics of right-sided EOCC patients in the training and validation cohorts for CSS | | | | |
| --- | --- | --- | --- | --- |
| Characteristic | All cohort  n=1922  *N* | Training cohort  n=1346  N(%) | Validation cohort  n=576  *N*(%) | *P*-value |
| sex |  |  |  | 0.349 |
| Female | 888 | 612 (45.5%)" | 276 (47.9%) |  |
| Male | 1034 | 734 (54.5%) | 300 (52.1%) |  |
| Histology |  |  |  | 0.708 |
| Non-specific adenocarcinoma | 1635 | 1150 (85.4%) | 485 (84.2%) |  |
| specific adenocarcinoma | 242 | 170 (12.6%) | 72 (12.5%) |  |
| other | 45 | 26 (1.9%) | 19 (3.3%) |  |
| Site |  |  |  | 0.192 |
| cecum | 873 | 603 (44.8%) | 270 (46.9%) |  |
| ascending Colon | 818 | 587 (43.6%) | 231 (40.1%) |  |
| Hepatic Flexure | 231 | 156 (11.6%) | 75 (13.0%) |  |
| Pathologic stage |  |  |  | 0.141 |
| Stage I-II | 784 | 534 (39.7%) | 250 (43.4%) |  |
| Stage III-IV | 1138 | 812 (60.3%) | 326 (56.6%) |  |
| Surgery of Primary Site |  |  |  | 0.168 |
| Yes | 1905 | 1331 (98.9%) | 574 (99.7%) |  |
| No | 17 | 15 (1.1%) | 2 (0.3%) |  |
| Reginal lymph node dissection |  |  |  | 0.641 |
| Yse | 1879 | 1314 (97.6%) | 565 (98.1%) |  |
| No | 43 | 32 (2.4%) | 11 (1.9%) |  |
| Radiation |  |  |  | 0.797 |
| Yes | 49 | 33 (2.5%) | 16 (2.8%) |  |
| No | 1873 | 1313 (97.5%) | 560 (97.2%) |  |
| Table S4 Continued | | | | |
| Chemotherapy |  |  |  | 0.490 |
| Yes | 1192 | 842 (62.6%) | 350 (60.8%) |  |
| No/unknown | 730 | 504 (37.4%) | 226 (39.2%) |  |
| Bone metastasis |  |  |  | 0.675 |
| Yes | 6 | 5 (0.4%) | 1 (0.2%) |  |
| No | 1916 | 1341 (99.6%) | 575 (99.8%) |  |
| Liver mestasis |  |  |  | 0.191 |
| Yes | 290 | 213 (15.8%) | 77 (13.4%) |  |
| No | 1632 | 1133 (84.2%) | 499 (86.6%) |  |
| Lung mestasis |  |  |  | 0.864 |
| Yes | 57 | 41 (3%) | 16 (2.8%) |  |
| No | 1865 | 1305 (97%) | 560 (97.2%) |  |
| Grade, n (%) |  |  |  | 0.161 |
| Well and moderate | 1447 | 1026 (76.2%) | 421 (73.1%) |  |
| poor | 475 | 320 (23.8%) | 155 (26.9%) |  |
| Pretreatment CEA level |  |  |  | 0.544 |
| negative | 1163 | 808 (60.0%) | 355 (61.6%) |  |
| elevated | 759 | 538 (40.0%) | 221 (38.4%) |  |
| Perineural invasion |  |  |  | 0.275 |
| Yse | 295 | 215 (16%) | 80 (13.9%) |  |
| No | 1627 | 1131 (84%) | 496 (86.1%) |  |
| Tumor size(mm) |  |  |  | 0.299 |
| <54.9 | 1064 | 756 (56.2%) | 308 (53.5%) |  |
| >54.9 | 858 | 590 (43.8%) | 268 (46.5%) |  |
| Table S4 Continued | | | | |
| Survival status |  |  |  | 0.656 |
| Alive | 1362 | 954 (71.1%) | 408 (70.8%) |  |
| Dead | 558 | 390 (28.9%) | 168 (29.2%) |  |
