# Supplementary material for: Development and validation of prognostic nomograms for early-onset colon cancer in different tumor locations: a population-based study
Source: BMC Gastroenterol. 2023 Oct 21;23:362. doi: 10.1186/s12876-023-02991-1 (PMC10590526; doi:10.1186/s12876-023-02991-1)
Supplement: Supplementary file 10 — Additional file 10: Table S5. Baseline characteristics of left-sided EOCC patients in the training and validation cohorts for CSS. [file 12876_2023_2991_MOESM10_ESM.docx]

| Table S5 Baseline characteristics of left-sided EOCC patients in the training and validation cohorts for CSS | | | | |
| --- | --- | --- | --- | --- |
| Characteristic | All cohort  n=2817  *N*(%) | Training cohort  n=1972  N(%) | Validation cohort  n=845  *N*(%) | *P*-value |
| sex |  |  |  | 0.567 |
| Female | 1472 | 1023 (51.9%) | 449 (53.1%) |  |
| Male | 1345 | 949 (48.1%) | 396 (46.9%) |  |
| Histology |  |  |  | 0.549 |
| Non-specific adenocarcinoma | 2638 | 1853 (94%) | 785 (92.9%) |  |
| specific adenocarcinoma | 148 | 99 (5%) | 49 (5.8%) |  |
| other | 31 | 20 (1%) | 11 (1.3%) |  |
| Site |  |  |  | 0.359 |
| Splenic Flexure | 215 | 159 (8.1%) | 56 (6.6%) |  |
| Descending Colon | 530 | 364 (18.5%) | 166 (19.6%) |  |
| Sigmoid Colon | 2072 | 1449 (73.5%) | 623 (73.7%) |  |
| Pathologic stage |  |  |  | 0.141 |
| Stage I-II | 1006 | 711 (36.1%) | 295 (34.9%) |  |
| Stage III-IV | 1811 | 1261 (63.9%) | 550 (65.1%) |  |
| Surgery of Primary Site |  |  |  | 0.763 |
| Yes | 2791 | 1955 (99.1%) | 836 (98.9%) |  |
| No | 26 | 17 (0.9%) | 9 (1.1%) |  |
| Reginal lymph node dissection |  |  |  | 0.671 |
| Yse | 2728 | 1912 (97%) | 816 (96.6%) |  |
| No | 89 | 60 (3.0%) | 29 (3.4%) |  |
| Radiation |  |  |  | 0.751 |
| Yes | 120 | 79 (4.0%) | 31 (3.7%) |  |
| No | 2697 | 1893 (96.0%) | 814 (96.3%) |  |
| Table S4 Continued | | | | |
| Chemotherapy |  |  |  | 0.768 |
| Yes | 1933 | 1357 (68.8%) | 576 (68.2%) |  |
| No/unknown | 884 | 615 (31.2%) | 269 (31.8%) |  |
| Bone metastasis |  |  |  | 0.999 |
| Yes | 16 | 11 (0.6%) | 5 (0.6%) |  |
| No | 2801 | 1961 (99.4%) | 840 (99.4%) |  |
| Brain metastasis |  |  |  | 0.331 |
| Yes | 5 | 4 (0.3%) | 1 (0.1%) |  |
| No | 2812 | 1967 (99.7%) | 844 (99.9%) |  |
| Liver mestasis |  |  |  | 0.192 |
| Yes | 511 | 345 (17.5%) | 166 (19.6%) |  |
| No | 2306 | 1627 (82.5%) | 679 (80.4%) |  |
| Lung mestasis |  |  |  | 0.752 |
| Yes | 97 | 66 (3.3%) | 31 (3.7%) |  |
| No | 2720 | 1906 (96.7%) | 814 (96.3%) |  |
| Grade, n (%) |  |  |  | 0.161 |
| Well and moderate | 2378 | 1647 (83.5%) | 731 (86.5%) |  |
| poor | 439 | 325 (16.5%) | 114 (13.5%) |  |
| Pretreatment CEA level |  |  |  | 0.668 |
| negative | 1608 | 1120 (56.8%) | 488 (57.8%) |  |
| elevated | 1209 | 852 (43.2%) | 357 (42.2%) |  |
| Perineural invasion |  |  |  | 0.204 |
| Yse | 580 | 419 (21.2%) | 161 (19.1%) |  |
| No | 2237 | 1553 (78.8%) | 684 (80.9%) |  |
|  |  |  |  |  |
| Table S4 Continued | | | | |
| Tumor size(mm) |  |  |  | 0.299 |
| <44.9 | 1955 | 1380 (70%)" | 575 (68%) |  |
| >44.9 | 862 | 592 (30%) | 270 (32%) |  |
| Survival status |  |  |  | 0.958 |
| Alive | 2051 | 1435 (69.9%) | 616 (72.9%) |  |
| Dead | 766 | 537 (29.1%) | 229 (27.1%) |  |
