# Supplementary material for: Development and validation of prognostic nomograms for early-onset colon cancer in different tumor locations: a population-based study
Source: BMC Gastroenterol. 2023 Oct 21;23:362. doi: 10.1186/s12876-023-02991-1 (PMC10590526; doi:10.1186/s12876-023-02991-1)
Supplement: Supplementary file 11 — Additional file 11: Table S6. Baseline characteristics of the transversed-sided EOCC patients in the training and validation cohorts for CSS. [file 12876_2023_2991_MOESM11_ESM.docx]

| Table S6 Baseline characteristics of the transversed-sided EOCC patients in the training and validation cohorts for CSS | | | | |
| --- | --- | --- | --- | --- |
| Characteristic | All cohort  n=531  *N*(%) | Training cohort  n=371  N(%) | Validation cohort  n=160  *N*(%) | *P*-value |
| sex |  |  |  | 0.454 |
| Female | 248 | 171(46.1%) | 77 (48.3%) |  |
| Male | 283 | 200 (53.9%) | 83 (51.7%) |  |
| Histology |  |  |  | 0.808 |
| Non-specific adenocarcinoma | 451 | 316 (85.4%) | 135 (84.4%) |  |
| specific adenocarcinoma | 66 | 45 (12.1%) | 21 (13.1%) |  |
| other | 14 | 10 (2.5%) | 4 (2.5%) |  |
| Pathologic stage |  |  |  | 0.543 |
| Stage I-II | 234 | 162 (43.8%) | 72 (45.6%) |  |
| Stage III-IV | 297 | 209 (56.2%) | 88 (54.4%) |  |
| Surgery of Primary Site |  |  |  | 0.732 |
| Yes | 527 | 368 (99.4%) | 159 (99.6%) |  |
| No | 4 | 3 (0.6%) | 1 (0.4%) |  |
| Reginal lymph node dissection |  |  |  | 0.368 |
| Yse | 520 | 363 (97.9%) | 157 (98.7%) |  |
| No | 11 | 8 (2.1%) | 3 (1.3%) |  |
| Radiation |  |  |  | 0.560 |
| Yes | 10 | 6 (1.3%) | 4 (1.9%) |  |
| No | 521 | 365 (98.6%) | 156 (98.1%) |  |
| Chemotherapy |  |  |  | 0.479 |
| Yes | 319 | 221 (59.8%) | 98 (61.8%) |  |
| No/unknown | 212 | 150 (30.2%) | 62 (38.2%) |  |
|  |  |  |  |  |
| Table S6 Continued | | | | |
| Bone metastasis |  |  |  | 0.448 |
| Yes | 4 | 2 (0.5%) | 2 (1.2%) |  |
| No | 527 | 369 (99.5%) | 158 (98.8%) |  |
| Liver metastasis |  |  |  | 0.345 |
| Yes | 81 | 55 (14.8%) | 26 (17.3%) |  |
| No | 450 | 316 (85.2%) | 134 (82.7%) |  |
| Lung metastasis |  |  |  | 0.759 |
| Yes | 9 | 5 (1.3%) | 4 (2.5%) |  |
| No | 522 | 366 (98.7%) | 156 (97.5%) |  |
| Grade, n (%) |  |  |  | 0.660 |
| Well and moderate | 404 | 281 (75.7%) | 123 (76.9%) |  |
| poor | 127 | 90 (24.3%) | 37 (23.1%) |  |
| Pretreatment CEA level |  |  |  | 0.383 |
| negative | 316 | 224 (60.4%) | 92 (57.5%) |  |
| elevated | 215 | 147 (39.6%) | 68 (42.5%) |  |
| Perineural invasion |  |  |  | 0.952 |
| Yse | 81 | 60 (16.2%) | 21 (13.1%) |  |
| No | 450 | 311 (83.8%) | 139 (86.9%) |  |
| Tumor size (mm) |  |  |  | 0.738 |
| <49.9 | 243 | 161 (53.5%) | 82 (51.5%) |  |
| >49.9 | 288 | 210 (56.5%) | 78 (48.5%) |  |
| Survival status |  |  |  | 0.853 |
| Alive | 357 | 247 (66.6%) | 110 (68.8%) |  |
| Dead | 174 | 124 (33.4%) | 50 (31.9%) |  |
