# Supplementary material for: Development and validation of prognostic nomograms for early-onset colon cancer in different tumor locations: a population-based study
Source: BMC Gastroenterol. 2023 Oct 21;23:362. doi: 10.1186/s12876-023-02991-1 (PMC10590526; doi:10.1186/s12876-023-02991-1)
Supplement: Supplementary file 12 — Additional file 12: Table S7. Univariate and multivariable Cox analysis for CSS of the right-sided EOCCs. [file 12876_2023_2991_MOESM12_ESM.docx]

| Table S7 Univariate and multivariable Cox analysis for CSS of the right-sided EOCCs | | | | | |
| --- | --- | --- | --- | --- | --- |
| Characteristics | Univariate analysis P-value | |  | Multivariable analysis P-value | |
|  | Hazard ratio (95% CI) |  |  | Hazard ratio (95% CI) |  |
| Sex |  |  |  |  |  |
| Female | Ref |  |  |  |  |
| Male | 1.143（0.969-1.350） | 0.113 | |  |  |
| Histology |  |  |  |  |  |
| Non-specific adenocarcinoma | Ref |  |  |  |  |
| Specific adenocarcinoma | 1.141（0.895-1.454） | 0.288 | |  |  |
| Others | 3.081（2.074-4.576） | 0.020 | | 1.263 （0.984-1.622） | 0.067 |
| Site |  |  |  |  |  |
| Cecum | Ref |  |  |  |  |
| Ascending colon | 1.114（0.853-1.454） | 0.428 | |  |  |
| Hepatic flexure | 0.916（0.696-1.205） | 0.532 | |  |  |
| Pathologic stage |  |  |  |  |  |
| I-II | Ref |  |  |  |  |
| III-IV | 13.572（9.637-19.115） | <0.001* | | 9.881（6.739-14.488） | <0.001* |
| Surgery of Primary Site |  |  |  |  |  |
| No | Ref |  |  |  |  |
| Yes | 0.103（0.062-0.170） | <0.001* | | 0.626（0.277-1.412） | 0.259 |
| Reginal lymph node dissection |  |  |  |  |  |
| No | Ref |  |  |  |  |
| Yes | 0.402（0.262-0.616） | 0.015* | | 1.084 (0.295-3.983) | 0.903 |
| Radiation |  |  |  |  |  |
| No | Ref |  |  |  |  |
| Yes | 2.542（1.747-3.691） | <0.001* | | 1.962（1.336-2.882） | 0.001* |
| Chemotherapy |  |  |  |  |  |
| No/unkniwn | Ref |  |  |  |  |
| Yes | 0.351（0.284-0.433） | <0.001* | | 0.544（0.428-0.690） | <0.001* |
| Bone metastasis |  |  |  |  |  |
| No | Ref |  | |  |  |
| Yes | 10.874（4.876-23.176） | <0.001* | | 1.067（0.455-2.500） | 0.881 |
| Liver metastasis |  |  |  |  |  |
| No | Ref |  | |  |  |
| Yes | 8.720（7.340-10.360） | <0.001* | | 3.957（3.235-4.841） | <0.001* |
| Lung metastasis |  |  | |  |  |
| No | Ref |  |  |  |  |
| Yes | 7.061（5.261-9.477） | <0.001* | | 2.210（1.614-3.027） | <0.001* |
| Grade |  |  |  |  |  |
| Poor | Ref |  |  |  |  |
| Well and moderate | 0.462（0.389-0.548） | <0.001* | | 0.611（0.508-0.735） | <0.001* |
|  |  |  |  |  |  |
|  |  |  |  |  |  |
| Table S7 (continued) | | | | | |
| Characteristics | Univariate analysis P-value | | | Multivariable analysis P-value | |
|  | Hazard ratio (95% CI) |  |  | Hazard ratio (95% CI) |  |
| Pretreatment CEA |  |  |  |  |  |
| Negative | Ref |  |  |  |  |
| Elevated | 3.140（2.645-3727） | <0.001* | | 1.477（1.218-1.792） | <0.001* |
| Perineural invasion |  |  |  |  |  |
| No | Ref |  |  |  |  |
| Yes | 3.140（2.618-3.767） | <0.001* | | 1.704（1.411-2.058） | <0.001* |
| Tumor size(mm) |  |  |  |  |  |
| <54.9 | Ref |  |  |  |  |
| >54.9 | 1.302（1.103-1.537） | 0.002* | | 1.306（1.098-1.552） | 0.003* |
| *Statistical signifcance | | | | | |
