# Supplementary material for: Development and validation of prognostic nomograms for early-onset colon cancer in different tumor locations: a population-based study
Source: BMC Gastroenterol. 2023 Oct 21;23:362. doi: 10.1186/s12876-023-02991-1 (PMC10590526; doi:10.1186/s12876-023-02991-1)
Supplement: Supplementary file 13 — Additional file 13: Table S8. Univariate and multivariable Cox analysis for CSS of the left-sided EOCCs. [file 12876_2023_2991_MOESM13_ESM.docx]

| Table S8 Univariate and multivariable Cox analysis for CSS of the left-sided EOCCs | | | | | |
| --- | --- | --- | --- | --- | --- |
| Characteristics | Univariate analysis P-value | | | Multivariable analysis P-value | |
|  | Hazard ratio (95% CI) |  |  | Hazard ratio (95% CI) |  |
| Sex |  |  |  |  |  |
| Female | Ref |  |  |  |  |
| Male | 0.879 (0.763-1.013) | 0.075 | |  |  |
| Histology |  |  |  |  |  |
| Non-specific adenocarcinoma | Ref |  |  |  |  |
| Specific adenocarcinoma | 1.471 (1.101-1.965) | 0.009 | | 1.883 (0.931-2.545) | 0.467 |
| Others | 4.615 (2.988-7.127) | 0.004 | | 2.578 (0.643-4.045) | 0.504 |
| Site |  |  |  |  |  |
| Cecum | Ref |  |  |  |  |
| Ascending colon | 0.856 (0.636-1.152) | 0.305 | |  |  |
| Hepatic flexure | 0.892 (0.688-1.157) | 0.390 | |  |  |
| Pathologic stage |  |  |  |  |  |
| I-II | Ref |  |  |  |  |
| III-IV | 6.324 (4.968- 8.050) | <0.001* | | 3.812 (2.845-5.108) | <0.001* |
| Surgery of Primary Site |  |  |  |  |  |
| No | Ref |  |  |  |  |
| Yes | 0.096 (0.063-0.145) | <0.001* | | 0.913 (0.339-2.462) | 0.858 |
| Reginal lymph node dissection |  |  |  |  |  |
| No | Ref |  |  |  |  |
| Yes | 0.502 (0.361-0.698) | <0.001* | | 0.531 (0.284-1.193) | 0.084 |
| Radiation |  |  |  |  |  |
| No | Ref |  |  |  |  |
| Yes | 1.510 (1.106-2.061) | 0.009* | | 1.199 (0.871-1.651) | 0.625 |
| Chemotherapy |  |  |  |  |  |
| No/unkniwn | Ref |  |  |  |  |
| Yes | 0.390 (0.321-0.473) | <0.001* | | 0.634 (0.505-0.798) | <0.001* |
| Bone metastasis |  |  |  |  |  |
| No | Ref |  |  |  |  |
| Yes | 10.693 (6.286-18.189) | <0.001* | | 3.006 (0.742-5.187) | 0.078 |
| Liver metastasis |  |  |  |  |  |
| No | Ref |  |  |  |  |
| Yes | 7.184 (6.219-8.297) | <0.001* | | 3.733 (3.150-4.423) | <0.001* |
| Lung metastasis |  |  |  |  |  |
| No | Ref |  |  |  |  |
| Yes | 5.372 (4.212-6.851) | <0.001* | | 1.766 (1.366-2.282) | <0.001* |
| Grade |  |  |  |  |  |
| Well and moderate | Ref |  |  |  |  |
| Poor | 2.214 (1.881-2.606) | <0.001* | | 1.905 (1.610-2.254) | <0.001* |
| Pretreatment CEA |  |  |  |  |  |
| Negative | Ref |  |  |  |  |
| Elevated | 3.791 (3.249-4.422) | <0.001* | | 1.763 (1.482-2.097) | <0.001* |
| Table S8 (continued) | | | | | |
| Characteristics | Univariate analysis P-value | | | Multivariable analysis P-value | |
|  | Hazard ratio (95% CI) |  |  | Hazard ratio (95% CI) |  |
| Perineural invasion |  |  |  |  |  |
| No | Ref |  |  |  |  |
| Yes | 2.730 (2.356-3.163) | <0.001* | | 1.510 (1.296-1.760) | <0.001* |
| Tumor size (mm) |  |  |  |  |  |
| <44.9 | Ref |  |  |  |  |
| >44.9 | 1.409 (1.217-1.633) | <0.001* | | 1.251 (0.965-1.454) | 0.054 |
| *Statistical signifcance |  |  |  |  |  |
