# Supplementary material for: Development and validation of prognostic nomograms for early-onset colon cancer in different tumor locations: a population-based study
Source: BMC Gastroenterol. 2023 Oct 21;23:362. doi: 10.1186/s12876-023-02991-1 (PMC10590526; doi:10.1186/s12876-023-02991-1)
Supplement: Supplementary file 14 — Additional file 14: Table S9. Univariate and multivariable Cox analysis for OS of the transverse-sided EOCCs. [file 12876_2023_2991_MOESM14_ESM.docx]

| Table S9 Univariate and multivariable Cox analysis for OS of the transverse-sided EOCCs | | | | | |
| --- | --- | --- | --- | --- | --- |
| Characteristics | Univariate analysis P-value | |  | Multivariable analysis P-value | |
|  | Hazard ratio (95% CI) |  |  | Hazard ratio (95% CI) |  |
| Sex |  |  |  |  |  |
| Female | Ref |  |  |  |  |
| Male | 0.816 (0.646-1.031) | 0.088 | |  |  |
| Histology |  |  |  |  |  |
| Non-specific adenocarcinoma | Ref |  |  |  |  |
| Specific adenocarcinoma | 1.010 (0.708-1.440) | 0.958 | |  |  |
| Others | 3.715 (2.164-6.376) | <0.001* | | 1.588（0.902-2.795） | 0.109 |
| Pathologic stage |  |  |  |  |  |
| I-II | Ref |  |  |  |  |
| III-IV | 18.066 (10.736-30.399) | <0.001* | | 12.042 (6.792-21.350) | <0.001* |
| Surgery of Primary Site |  |  |  |  |  |
| No | Ref |  |  |  |  |
| Yes | 2.031 (0.310-5.130) | 0.064 | |  |  |
| Reginal lymph node dissection |  |  |  |  |  |
| No | Ref |  |  |  |  |
| Yes | 0.293 (0.168-0.511) | <0.001* | | 0.442 (0.181-0.770) | 0.032* |
| Radiation |  |  |  |  |  |
| No | Ref |  |  |  |  |
| Yes | 2.957 (1.617-5.404) | <0.001* | | 1.824 (0.978-3.401) | 0.059 |
| Chemotherapy |  |  |  |  |  |
| No/unkniwn | Ref |  |  |  |  |
| Yes | 0.319 (0.236-0.431) | <0.001* | | 0.526 (0.283-0.976) | 0.042* |
| Bone metastasis |  |  |  |  |  |
| No | Ref |  |  |  |  |
| Yes | 7.394 (0.725-19.880) | <0.001* | | 1.140（0.403-3.221） | 0.805 |
| Liver metastasis |  |  |  |  |  |
| No | Ref |  |  |  |  |
| Yes | 8.735 (6.873-11.101) | <0.001* | | 3.723 (2.809-4.935) | <0.001* |
| Lung metastasis |  |  |  |  |  |
| No | Ref |  |  |  |  |
| Yes | 8.259 (5.563-12.262) | <0.001* | | 2.598 (1.703-3.963) | <0.001* |
| Grade |  |  |  |  |  |
| Well and moderate | Ref |  |  |  |  |
| Poor | 2.569 (2.022-3.263) | <0.001* | | 1.875 (1.445-2.434) | <0.001* |
| Pretreatment CEA level |  |  |  |  |  |
| Negative | Ref |  |  |  |  |
| Elevated | 2.993 (2.350-3.812) | <0.001* | | 1.354 (1.033- 1.775) | 0.028 |
| Perineural invasion |  |  |  |  |  |
| No | Ref |  |  |  |  |
| Yes | 3.677 (2.866-4.716) | <0.001* | | 1.989 (1.525- 2.595) | <0.001* |
|  |  |  |  |  |  |
|  |  |  |  |  |  |
| Table 6 (continued) | | | | | |
| Characteristics | Univariate analysis P-value | | | Multivariable analysis P-value | |
|  | Hazard ratio (95% CI) |  |  | Hazard ratio (95% CI) |  |
| Tumor size(mm) |  |  |  |  |  |
| <54.9 | Ref |  |  |  |  |
| >54.9 | 1.458 (1.154-1.843) | 0.002* | | 1.578 (1.235-2.015) | <0.001* |
| *Statistical signifcance | | | | | |
